# Incidence and intensity of catastrophic health expenditure and impoverishment among the elderly: an empirical evidence from India

**DOI:** 10.1038/s41598-024-55142-1

**Published:** 2024-07-10

**Authors:** Fahimuddin Ahmad, Pratap Chandra Mohanty

**Affiliations:** Department of Humanities and Social Sciences, Indian Institute of Technology Roorkee, Roorkee, Uttarakhand 247667 India

**Keywords:** Health care, Health occupations

## Abstract

World health statistics (2022) report that about 1.4 billion people have incurred catastrophic health expenditure (CHE), and half of its population have pushed into extreme poverty due to healthcare payments. The elderly population faces a higher risk of ill health, and seeking treatment reflects in high out-of-pocket health spending (OOPHS) that results in CHE and further impoverishment. This study aims to investigate the incidence and intensity of OOPHS, CHE, and impoverishment among the older adults in India. Data utilizes from the 75th round of the national sample survey (NSS) based on household social consumption: health (schedule 25.0), 2018. The incidence and intensity of CHE and impoverishment among older adults in India estimated by using standard measures. The older adults spend about 17.4% of household consumption expenditure on healthcare services. The poorest older adults are spending the highest share of consumption expenditure (24.8%) on healthcare among economic quantiles. Similarly, the elderly from rural (20.3%), male (18.4%), scheduled castes (21.5%), and Hinduism (17.9%) show a larger share of consumption expenditure on healthcare services. The incidence and intensity of CHE among older adults are 46.5% and 16.1% at 10% threshold level of household consumption expenditure, respectively. However, as the threshold level rises the incidence and intensity of CHE decline. The estimates of normalized poverty gap using the poverty line of Tendulkar committee as well as Rangarajan committee show that the intensity of impoverishment among older adults are 56.4% and 57.9% respectively, which is quite high. Financial protection along with vision might mitigate the risk of CHE and impoverishment among older adults in India.

## Introduction

Globally, around 1.4 billion population has incurred catastrophic health expenditure (CHE), and almost 70 million population has been pushed into extreme poverty, while a further 435 million population has been pushed deeper into extreme poverty due to out-of-pocket health spending (OOPHS)^1^. National health accounts (2023) estimates OOPHS as a percentage of total health expenditure in India is 47.1%^2^, which is quite high. Out-of-pocket healthcare financing is still a challenge to cope up within India. The continuous increasing cost of healthcare services lead to financially burden of the households, which results in CHE^3^. The previous findings reveal several socioeconomic and health determinants that affect households’ CHE^4^, resulting in impoverishment. The determinants of CHE such as economic status, hospitalization, a disabled person in the household, a person with chronic illness, presence of older adults in households, presence of children in the family are the common determinants associated with CHE^4–9^. The ‘presence of older adults among household members’ as an explanatory variable significantly impacts household’s CHE and impoverishment^10–13^.

The older adults are one of the most vulnerable groups in terms of health and healthcare utilization. Aging is a growing concern nowadays in the world. Globally, the elderly population aged 60 and above increased from one billion in 2020 to 1.4 billion, and by 2050, it is expected to double (WHO, 2021). According to the World Health Organization (2021), 80 percent of the elderly population will be living in low-and middle-income countries by 2050. The share of the elderly population aged 60 and above in India has been projected to increase from 8.6 percent in 2011 to 10.1 percent in 2021 and is expected to rise by 13.1 percent in 2031 (MOSPI, 2021). The elderly population faces a higher risk of multimorbidity. Without the proper financial support along with vision, their quality of life will be severely compromised^14^. Health is considered as an important indicator in measuring human development index (HDI), and sustainable development goal three (SDG 3) too focus on “ensuring a healthy life and promoting well-being for all ages,” which makes people abilities to develop more proficiency in various social, economic, and productive fields^15^. But to avail the healthcare service is very challenging in the developing nations^16^. In the recent covid-19 pandemic, we witnessed how the healthcare system crumpled in India^17^. Health expenditure during the covid-19 crisis increases multifold^18^. Therefore, lousy health increases the risk of bankruptcy in vulnerable households.

Furthermore, the affordability and accessibility of healthcare are not equally enjoyed by all socioeconomic population strata within a society^19^. This inequality in healthcare utilization persists due to both social-demographic and economic conditions. Poor healthcare infrastructure, long waiting queue, non-availability of healthcare professionals in remote area, quackery, major distance of healthcare facilities from home, negligible or low income, high costs of medical tests, low insurance coverage or private healthcare for huge profits are some of the barriers of healthcare utilization^19–21^. There is a tendency to shift more toward private health facilities because of the poor condition of the public health system^22^. Older men and persons with chronic diseases seeking treatment in private healthcare services have a higher incidence of CHE and impoverishment in India^23^. Moreover, a case study from Lucknow, India, based on CHE among the elderly population, reports that the risk of facing CHE is higher among the lower-income group^24^. The vulnerable elderly population refrain from utilizing healthcare services due to financial constraints. The household spends on healthcare from their pocket^25^, which could be the possibility of frequent outpatient services and low health insurance coverage. After analyzing the 75th round of National Sample Survey (NSS) dataset which utilized in this study, we found that about 81% of older adults do not have any type of health insurance coverage in India.

The effect of CHE and further impoverishment among older adults can be grievous, which can lead to barriers in accessing healthcare services, degradation in quality of life, financial hardships, and trap into chronic poverty. Overall, these issues lead to a challenging economic and social development for the elderly population as well as society to a greater extent. The study highlights the essence of in-depth investigation into the prevalence of the elderly population facing the risk of financial catastrophe and impoverishment due to healthcare payments in different socio-economic strata. This study contributes to the existing literature on these current issues and suggests to policymakers and other stakeholders how to cope with financial hardship due to OOPHS among the elderly population.

## Methods

### Data and variable

Cross-sectional data is used for the study from the 75th round of the NSS dataset based on household social consumption: health (Schedule 25.0), conducted by the national statistical office between July-2017 and June-2018. The NSS data is nationally representative and covers all the Indian states and Union territories. It has used a standard questionnaire that covers a wide range of quarries on households as well as individual characteristics, demographic, in-patient and out-patient treatment and expenses, and economic independence and health status of those aged 60 and above. A multi-stage stratified sampling design is adopted for the 75^th^ round of NSS. In rural areas, the first stage units (FSU) are the Census villages vis-à-vis urban frame survey (UFS) blocks in urban areas. Households are the ultimate stage units (USU) in both regions. In the case of large FSUs, one intermediate stage of sampling is the selection of two sub-blocks/hamlet groups from each urban UFS/rural FSU. A total of 1,13,823 households and 5,55,352 members of the households are included in the survey from every district of the country. We utilize the sample of the elderly population in the survey, which consists of 42,762 individuals. In this paper, A total of 18,901 elderly population were hospitalized in 365 days, whereas 11,084 elderly population reported ailments in the last 15 days period. With all the information on medical expenses, we have included a sample of 22,452 elderly population either hospitalized in a year or reported ailment in the last 15 days or both (i.e., converted the 15 days medical expenses into a 365-day period for outpatient services). The details of individual characteristics have been described in the NSS 2017-18 report^26^.

The socioeconomic and demographic characteristics of older adults are taken as follows: Age divided into three groups (60–69 years, 70–79 years, and 80 years and above), economic quantile divided into five groups (poorest, poor, middle, rich, and richest), sector (rural and urban), sex (Male and female), social groups (Schedule Tribe (ST), Schedule Caste (SC), Other Backward Class (OBC), and others), religion (Hinduism, Islam, Christian, and others), and insurance coverage (government, private, others, and none).

### Estimating catastrophic health expenditure (CHE)

The methodology for estimating CHE and impoverishment developed by Ke Xu which is adopted by World Health Organization (WHO) applied in this study^27^. CHE is defined as if OOPHS surpasses a certain threshold level of annual income or expenditure^28,29^. However, the data does not have information on household income; therefore, we are taking household usual consumption expenditure as a proxy income variable. Since poorer households spend most of their earnings on basic needs. Thus, they may not seek healthcare services that are unable to meet CHE hence underestimating the burden of OOPHS. The literature suggests measuring CHE if OOPHS surpasses the given threshold of household non-subsistence expenditure or capacity to pay^29^. Capacity to pay or non-subsistence expenditure (*Y*) is calculated as the difference between total household usual consumption expenditure (*X*) and subsistence expenditure (*S*).

$$Y=X-S$$

In this study, we use both total household usual consumption expenditure and non-subsistence expenditure methods to estimate CHE.

$$CHE=\frac{OP}{Y} or \frac{OP}{X}\ge Z$$

where $CHE, \;OP\;{\text{ and}}\; Z$ represents catastrophic health expenditure, out-of-pocket health spending, and threshold level, respectively.

Throughout the literature, there is not any well-defined threshold level. It ranges from 5 to 40 percent^4^. Most commonly threshold level is taken as 10 percent of total household expenditure and 40 percent of non-subsistence expenditure. Thus, we have also used additional threshold levels (20%, 30%, & 40% at subsistence expenditure and 20%, & 30% at non-subsistence expenditure) to enquire about potential outcomes and robustness checks. Additionally, we measure the estimate of inpatient and outpatient CHE separately which is presented in table Appendix 1.

### Estimating the incidence and intensity of catastrophic health expenditure

Headcount is used to measure the incidence (H) of CHE. It is calculated by the proportion of households that obtained CHE and is calculated as follows:

$$H=\frac{1}{N}\sum_{i=1}^{N}{E}_{i}$$

where *H* and *N* represent the incidence of CHE and the sample size, respectively. *E* is an indicator variable such as ${E}_{i}=1$ if $\frac{{OP}_{i}}{{X}_{i}}\ge Z$; and 0, otherwise.

Headcount is not sufficient to reflect the magnitude by which households surpass the threshold level. Hence, we use overshoot to capture the intensity (O) of CHE. It expresses the average degree of CHE households to which health expenditure surpasses the given threshold level, Z. The overshoot is calculated by the formula given below.

$${O}_{i}={E}_{i }\left[\left(\frac{{OP}_{i}}{{X}_{i}}\right)-Z\right]$$

Then, the mean of the overshoot is as follows:

$$O=\frac{1}{N}\sum_{i=1}^{N}{O}_{i}$$

where O represents the intensity of CHE.

The mean positive overshoot (MPO) is estimated to capture the intensity of the occurrence of CHE, which is defined as the ratio of overshoot and headcount.

$$MPO=\frac{O}{H}$$

We also use concentration indices, ${CI}_{E}$ and ${CI}_{O}$, for ${E}_{i}$ and ${O}_{i}$ respectively, to estimate the distribution of CHE with respect to household usual consumption expenditure since both the estimates headcount and overshoot are unaffected to the distribution of CHE. The range of concentration index is between $- 1 \;\;{\text{and}}\;\; + 1$. Positive value of ${CI}_{E}$ denotes the better-off households are more likely to surpass the given threshold level. Whereas the negative value of ${CI}_{E}$ represents the worse-off households are more likely to surpass the given threshold level. Similarly, if ${CI}_{O}$ is positive, the intensity of CHE is concentrated among the rich, and if negative, then the poor.

We use the weighted headcount and overshoot estimates to see the effect of OOPHS when the different weights have been assigned to the households based on their expenditure level. The weighted headcount and overshoot estimates are calculated as follows:

$${H}_{weight}=H\times \left(1-{CI}_{E}\right)$$

$${O}_{weight}=O\times \left(1-{CI}_{O}\right)$$

If the ${CI}_{E}$ is negative, the weighted headcount (${H}_{weight}$) is greater than the headcount (*H*), and the same explanation for the weighted overshoot.

### Health expenditure and impoverishment effect

Impoverishment is defined as a household or individual being pushed into poverty due to higher OOPHS. The OOPHS is not considered in the poverty estimation. We can estimate the impoverishment effect by the difference between poverty level before and after OOPHS. First, we obtain poverty headcount $(PH)$ before health expenditure, which gives the proportion of the population living below poverty before health expenditure.

$${PH}_{before}= \frac{{\sum }_{i=1}^{N}{S}_{i}\times {P}_{i}^{before}}{{\sum }_{i=1}^{N}{S}_{i}}$$

$${\text{Where}} \;\;P_{i}^{before} = \left\{ {\begin{array}{*{20}l} {1;} \hfill & {if\; x_{i} \le PL} \hfill \\ {0;} \hfill & { otherwise} \hfill \\ \end{array} } \right.$$

where $x_{i} ,$ $PL, \;S_{i} , \;{\text{and}}\; N$ denote the per capita household usual consumption expenditure, poverty line, household size, and total sample, respectively.

Now we can measure the poverty gap (PG) before health expenditure as below, which shows the aggregate deficit from the poverty line.

$${PG}_{i}^{before}= {P}_{i}^{before}\left(PL-{x}_{i}\right)$$

We can estimate the average poverty gap (${PG}_{mean}$) before health expenditure based on the above equation as follows:

$${PG}_{mean}^{before}=\frac{{\sum }_{i=1}^{N}{S}_{i}\times {PG}_{i}^{before}}{{\sum }_{i=1}^{N}{S}_{i}}$$

The normalized poverty gap (${PG}_{n}$) before health expenditure can be estimated by the ratio of poverty gap (${PG}_{i}^{before}$) and poverty line ($PL$).

$${PG}_{n}^{before}=\frac{{PG}_{i}^{before}}{PL}$$

Similarly, we can estimate poverty headcount, poverty gap, and normalized poverty gap after health expenses by replacing subscripts from before to after. STATA 17 is used for data analysis.

### Poverty line

A poverty line must be identified to calculate all the above poverty estimates. The poverty line defined as an essential consumption expenditure (i.e., food and non-food expenditure) needed to maintain a minimal acceptable of living standards. The two Indian national poverty line measures used in this study. The first is set by the Tendulkar committee, which is Indian Rupees (INR) 816 per month per person for rural areas and INR 1000 per month per person for urban areas. Another is set by the Rangarajan committee, which is INR 972 and INR 1407 per month per person for rural and urban areas, respectively. For more details, see the reports by the Tendulkar and the Rangarajan Committee^30,31^.

## Results

Table 1 shows the descriptive characteristics of older adults in India. The mean age of older adults’ population is 67.5 years, and most older adults (66.1%) fall into the 60–69 years age groups. In numbers it is quite high. The economic quantile divided into five groups from poorest to richest. The proportion of older adults residing in rural areas is twice as high as urban areas. The proportion of female counterpart is slightly higher than male counterpart. In social groups, Other Backward Classes (OBC) consists of the highest population (42.3%), followed by Others (34.1%), Schedule Castes (17.4%), Schedule Tribes (6.2%). Most of the elderly population belongs to Hinduism. 81.1% of older adults do not have any types of insurance coverage. Only 16.4% and 2.3% of older adults have government and private health insurance schemes, respectively.

**Table 1:** Elderly population socioeconomic & demographic characteristics.

Elderly characteristics	India	Sample size (N)
Mean age in years (S.D.)	67.5 (6.8)	42,762
Age group (%)		
60–69	66.1	27,769
70–79	25.9	11,235
80 and above	8.0	3758
Economic quantile (%)		
Poorest	21.9	7191
Poor	19.4	7148
Middle	17.6	7508
Rich	19.1	9,249
Richest	21.9	11,666
Sector (%)		
Rural	67.1	23,599
Urban	32.9	20,858
Sex (%)		
Male	49.1	21,902
Female	50.9	20,858
Social groups (%)		
ST	6.2	3913
SC	17.4	6,133
OBC	42.3	16,519
Others	34.1	16,197
Religion (%)		
Hinduism	83.3	33,243
Islam	10.6	4934
Christian	2.9	2573
Others	3.2	2012
Insurance coverage (%)		
Government	16.4	7234
Private	2.3	1452
Others	0.2	189
None	81.1	33,887

*Source* Author's computation using NSSO 75th round, 2018.

*S.D.* Standard Deviation.

### Out-of-pocket health spending on elderly inpatient services, outpatient services, and both

In this paper, we measured the OOPHS, CHE, and impoverishment of the Indian elderly population. Hospitalization expenses include package components, doctor/surgeon fees, medicines, diagnostic tests, bed charges, other medical expenses, transport, and other non-medical expenses during the medical process in a year. The outpatient services cover all the above costs except the package component for the last 15 days. We have added the medical insurance premium and subtracted the medical reimbursement from the total medical expenditure for both inpatient and outpatient services to estimate the OOPHS on elderly health.

**Figure 1:**
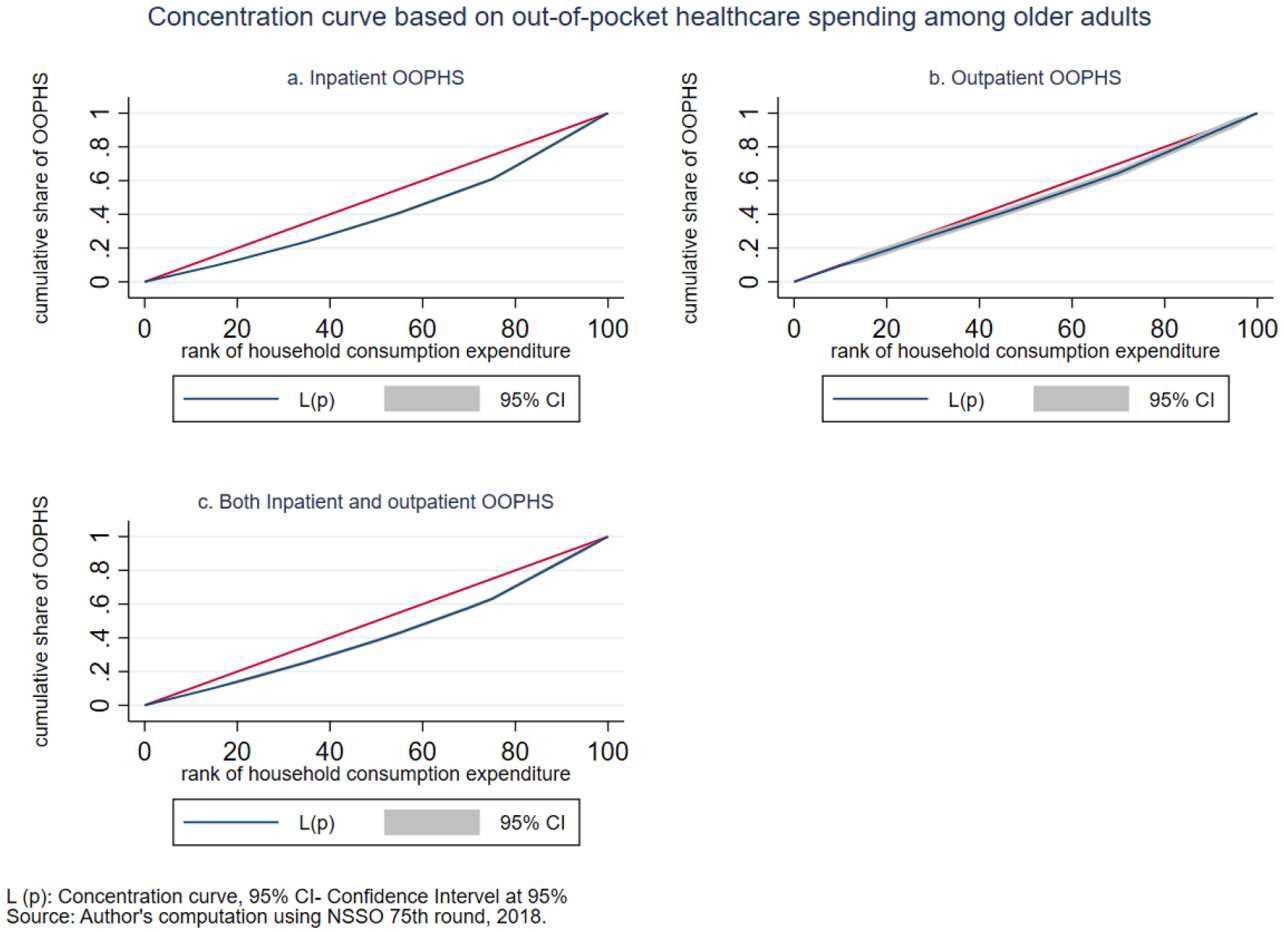
Concentration curve based on out-of-pocket healthcare spending among older adults.

Table 2 shows that the average OOPHS on elderly health for hospitalization (inpatient) and for reporting ailment in the last 15 days (outpatient) is INR 23,234 and INR 785, respectively. After adjusting outpatient expenses, the average OOPHS for both outpatient and inpatient is INR 23,459, which is approximately equal to the inpatient OOPHS. In the economic quantile group, the share of inpatient OOPHS increases as the economic quantile moves from poorest to richest groups. The outpatient OOPHS increases into lower quantiles but declines in the middle quantile, which is the lowest, and further increases in the upper quantiles, and a similar pattern is observed for both inpatient and outpatient OOPHS. The OOPHS on elderly health is higher for males in urban areas than for females in rural areas in all types of medical expenditures. In the social group, the inpatient OOPHS is higher for the general category (others) and lowest for schedule tribes (ST), and the outpatient OOPHS is higher in schedule caste (SC). For religion, inpatient OOPHS is higher among Christian and lowest in Islam, but outpatient OOPHS is lowest in Christian. Inpatient OOPHS is the highest for private health insurance coverage. Moreover, OOPHS is substantially high in all types of health insurance coverage. The concentration indices reflect that the elderly belonging to the richest households were more likely to report OOPHS on healthcare services (CI = 0.204 for inpatient services; 0.036 for outpatient services; and 0.101 for both), and all these differences are significant for all categories (*p* < 0.001). Similarly, the concentration curves in Fig. 1 show the inequality of OOPHS on healthcare services between the richest and poorest quantile of the elderly population.

**Table 2:** Mean out-of-pocket health spending on elderly health in Indian rupees.

	Inpatient OOPHS			Outpatient OOPHS			Both (inpatient & outpatient) OOPHS		
	Total obs.	Mean (INR)	S.D	Total obs.	Mean (INR)	S.D	Total obs.	Mean (INR)	S.D
	18,901	23,234.7	56,047.9	11,084	785.4	1650.1	22,452	23,459.7	51,721.2
Economic quantile									
Poorest	2832	14,105.4	37,437.8	1200	689.0	1011.8	3217	17,666.5	32,849.0
Poor	3030	16,162.4	36,871.3	1499	808.2	2111.5	3500	20,947.1	50,791.6
Middle	3338	18,544.6	40,181.7	1766	681.8	1398.9	3854	19,974.4	40,328.3
Rich	4306	24,887.2	49,926.2	2567	811.9	1438.8	5090	24,692.0	46,308.8
Richest	5395	37,550.2	84,708.0	4052	853.2	1851.4	6791	29,529.2	67,968.1
Sector									
Rural	10,146	19,003.5	45,779.7	5457	753.9	1671.3	11,867	21,242.0	48,067.1
Urban	8755	30,762.8	70,094.1	5627	832.9	1616.4	10,585	27,025.8	56,927.9
Sex									
Male	14,765	23,560.5	53,575.3	7527	836.7	1718.7	16,368	25,134.9	52,517.5
Female	4134	22,118.9	63,870.0	3555	680.1	1494.6	6082	19,695.6	49,683.6
Social group									
SC	2664	14,618.4	34,315.6	1460	923.4	2267.1	3121	22,599.3	53,647.5
ST	1593	10,121.1	21,761.4	550	614.4	1008.4	1772	14,754.1	27,061.0
OBC	7314	20,769.0	43,605.4	4133	711.8	1341.2	8552	21,401.5	41,953.5
Others	7330	31,463.3	75,069.7	4941	815.9	1660.5	9007	26,941.1	61,021.8
Religion									
Hinduism	14,505	23,475.1	55,657.1	8278	791.6	1666.6	17,170	23,417.4	51,210.1
Islam	2246	18,091.0	47,933.8	1538	736.1	1305.1	2717	21,586.0	43,262.7
Christian	1265	30,071.6	76,729.6	725	670.9	2153.1	1509	25,069.0	76,281.7
Others	885	28,138.9	58,702.5	543	948.8	1463.3	1056	28,387.0	49,319.4
Insurance coverage									
Government	3486	16,438.2	42,482.1	2431	696.4	2093.9	4214	19,788.4	53,920.8
Private	708	308,177.4	56,258.1	571	787.5	1109.3	892	25,844.5	42,541.8
Others	118	25,564.4	49,871.9	79	735.4	908.4	134	29,423.2	43,845.0
None	14,589	24,645.7	58,851.2	8003	811.5	1522.8	17,212	24,312.3	51,502.9
Concentration Index (s.e.)	18,901	0.205 (0.017)*		11,084	0.037 (0.025)		22,452	0.102 (0.018)*	

*Source* Author's computation using NSSO 75th round, 2018.

*p-value < 0.01, *s.e.* standard error, *S.D.* standard deviation.

**Figure 2:**
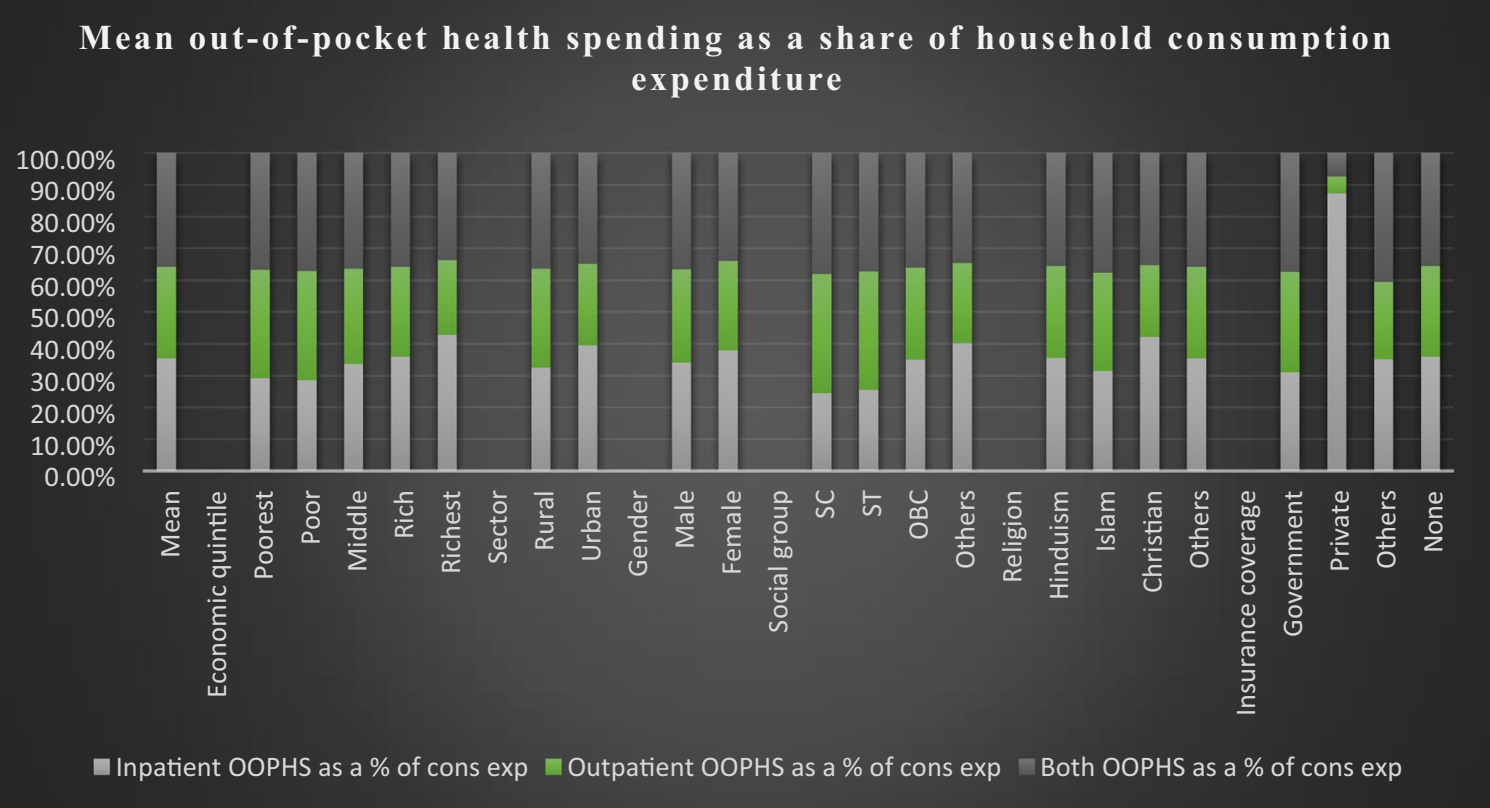
Mean out-of-pocket health spending as a share of household consumption expenditure.

Figure 2 shows the mean OOPHS as a share of household consumption expenditure on elderly healthcare. The overall mean OOPHS as a percentage of consumption expenditure among older people is 17.39%, with which inpatient and outpatient consist of 17.22% and 13.97%, respectively. The overall poorest quantile shows the highest share of consumption expenditure on healthcare (24.8%) compared to the richest quantile (12.77%). Similarly, the poorest quantile spends a significant amount on inpatient (19.8%) and outpatient (23.21%) services compared to the richest quantile, i.e., for inpatient (16.24%) and outpatient (8.85%). The elderly population living in rural areas spends a more considerable proportion of consumption expenditure on healthcare in comparison with the urban elderly population, i.e., 20.33% and 13.71%, respectively. There is also a higher difference between male (18.42%) and female (14.75%) spending on healthcare services. In the social group, the scheduled caste (21.52%) spends a large share of consumption expenditure on healthcare compared to others (16%). The spending on outpatient services (21.11%) is much higher than inpatient services (13.92%) among scheduled castes. Christian spends the highest amount, about 19.23%, for inpatient services but the lowest for outpatient services, i.e., 10.29%, compared to other religious groups. OOPHS due to private health insurance coverage has the highest for inpatient services but lowest for outpatient and both compared to government health insurance coverage. Those older adults who do not have any type of health schemes spend 18.6% of consumption expenditure on healthcare services.

### Incidence and intensity of catastrophic health expenditure

Tables 3, 4, 5, 6, 7 and 8 present the incidence and intensity of CHE of the elderly population, respectively. The results show the CHE as a share of household usual consumption expenditure (threshold level: 10%, 20%, 30%, 40%) and as a share of non-subsistence expenditure (threshold level: 20%, 30%, 40%). Catastrophic headcount shows a decreasing trend as the threshold level increases. 46.51% of the elderly population describes total OOPHS exceeding 10% of total household usual consumption expenditure, whereas after increasing the threshold level to 40%, the headcount reduces to 13.71%. Similarly, 42.22% (44.2%) of the elderly population reports total OOPHS exceeding 20% of non-subsistence expenditure, as per the Tendulkar committee poverty line (as per the Rangarajan committee poverty line). At the 40% threshold level, the catastrophic headcount reduces to 26.27% (28.82%). If we compare the result for OOPHS exceeding 40% of total household usual consumption expenditure and non-subsistence expenditure, the catastrophic headcount increases from 13.71% to 26.27% (28.82% for Rangarajan committee poverty line). The catastrophic headcount of the elderly population among the poorest quantile, residing in rural areas, being male, belonging from schedule caste/tribes, and having no insurance coverage are higher than their respective counterparts.

**Table 3:** Incidence of catastrophic health expenditure of elderly population.

CHE as a share of household usual consumption expenditure (%)									
Threshold (%)	Total obs.	10%		20%		30%		40%	
Headcount (%)									
		Mean (%)	S.D	Mean (%)	S.D	Mean (%)	S.D	Mean (%)	S.D
	22,452	46.51	0.49	28.04	0.44	18.62	0.38	13.71	0.34
Economic quantile									
Poorest	3217	55.54	0.49	37.35	0.48	28.35	0.45	22.43	0.41
Poor	3500	51.58	0.49	34.36	0.47	20.42	0.40	14.76	0.35
Middle	3854	47.23	0.49	27.86	0.44	18.42	0.38	12.23	0.32
Rich	5090	44.15	0.49	25.02	0.43	17.06	0.37	12.5	0.33
Richest	6,791	39.43	0.48	21.07	0.40	13.1	0.33	9.73	0.29
Sector									
Rural	11,867	49.79	0.49	31.32	0.46	20.89	0.40	15.51	0.36
Urban	10,585	41.22	0.49	22.78	0.41	14.97	0.35	10.81	0.31
Sex									
Male	16,368	47.92	0.49	29.06	0.45	19.02	0.39	13.94	0.34
Female	6082	43.33	0.49	25.75	0.43	17.72	0.38	13.18	0.33
Social group									
SC	3121	50.85	0.49	32.79	0.46	21.64	0.41	15.22	0.35
ST	1772	48.97	0.49	31.99	0.46	21.95	0.41	14.08	0.34
OBC	8552	45.96	0.49	27.44	0.44	18.12	0.38	13.09	0.33
Others	9007	44.91	0.49	26.16	0.43	17.45	0.37	13.65	0.34
Religion									
Hinduism	17,170	47.20	0.49	28.65	0.45	19.35	0.39	14.38	0.35
Islam	2717	46.15	0.49	25.47	0.43	14.46	0.35	9.86	0.29
Christian	1509	40.60	0.49	23.61	0.42	14.37	0.35	9.36	0.29
Others	1056	39.43	0.48	28.06	0.44	20.70	0.40	16.30	0.36
Insurance coverage									
Government	4214	41.90	0.49	24.19	0.42	16.46	0.37	10.53	0.30
Private	892	34.09	0.47	14.72	0.35	11.30	0.31	8.85	0.28
Others	134	45.82	0.49	31.08	0.46	20.45	0.40	16.84	0.37
None	17,212	48.36	0.49	29.72	0.45	19.56	0.39	14.79	0.35
Rank-weighted headcount (%)	22,452	52.58	0.56	32.74	0.52	21.92	0.45	16.16	0.40

Concentration Index headcount	Total obs.	Index value	s.e.*	Index value	s.e.*	Index value	s.e.*	Index value	s.e.*
	22,452	− 0.130	0.021*	− 0.167	0.023*	− 0.177	0.027*	− 0.178	0.031*

*Source* Author's computation using NSSO 75th round, 2018.

*p-value < 0.01, *s.e.* standard error, *S.D.* standard deviation.

**Table 4:** Intensity of catastrophic health expenditure of elderly population.

CHE as a share of household usual consumption expenditure (%)									
Threshold (%) Overshoot (%)	Total obs.	10%		20%		30%		40%	
		Mean (%)	S.D	Mean (%)	S.D	Mean (%)	S.D	Mean (%)	S.D
	22,452	16.10	0.55	12.53	0.53	10.26	0.52	8.67	0.50
Economic quantile									
Poorest	3217	25.90	0.71	21.49	0.69	18.26	0.67	15.71	0.65
Poor	3500	19.55	0.64	15.34	0.62	12.65	0.61	10.98	0.59
Middle	3854	14.81	0.51	11.32	0.50	9.04	0.48	7.55	0.47
Rich	5090	13.87	0.51	10.51	0.49	8.48	0.48	7.03	0.46
Richest	6791	10.56	0.41	7.77	0.39	6.13	0.37	5.02	0.36
Sector									
Rural	11,867	18.11	0.58	14.23	0.57	11.68	0.55	9.90	0.53
Urban	10,585	12.85	0.49	9.80	0.47	7.97	0.45	6.70	0.44
Sex									
Male	16,368	15.64	0.51	11.97	0.49	9.64	0.48	8.01	0.46
Female	6,082	17.12	0.63	13.78	0.61	11.63	0.59	10.10	0.58
Social group									
SC	3121	21.89	0.84	17.85	0.83	15.27	0.81	13.49	0.80
ST	1772	15.40	0.39	11.58	0.37	8.91	0.35	7.17	0.33
OBC	8552	15.95	0.49	12.42	0.47	10.17	0.45	8.63	0.44
Others	9007	13.82	0.45	10.44	0.43	8.32	0.41	6.79	0.40
Religion									
Hinduism	17,170	16.38	0.53	12.73	0.51	10.40	0.49	8.75	0.48
Islam	2717	12.11	0.38	8.81	0.36	6.77	0.34	5.61	0.33
Christian	1509	14.40	0.59	11.37	0.57	9.55	0.56	8.46	0.54
Others	1056	24.63	1.10	21.32	1.08	18.95	1.07	17.09	1.06
Insurance coverage									
Government	4214	14.09	0.55	10.95	0.53	9.01	0.52	7.71	0.50
Private	892	8.88	0.26	6.57	0.23	5.29	0.21	4.24	0.19
Others	134	12.96	0.28	9.35	0.26	6.79	0.23	4.98	0.21
None	17,212	17.00	0.56	13.26	0.54	10.85	0.53	9.16	0.51
Rank-weighted overshoot (%)	22,452	18.92	0.65	15.04	0.64	12.46	0.63	10.63	0.61
Mean positive overshoot (%)	22,452	16.1	0.55	12.53	0.53	10.26	0.52	8.67	0.50

Concentration Index overshoot	Total obs.	Index value	s.e.*	Index value	s.e.*	Index value	s.e.*	Index value	s.e.*
	22,452	− 0.175	0.037*	− 0.200	0.046*	− 0.214	0.055*	− 0.225	0.063*

*Source* Author's computation using NSSO 75th round, 2018.

*p-value < 0.01, s.e.- standard error, S.D.- standard deviation.

**Table 5:** Incidence of catastrophic health expenditure of elderly population (Tendulkar).

CHE as a share of household usual consumption expenditure (%)							
Threshold (%)	Total obs.	20%		30%		40%	
Headcount (%)							
		Mean (%)	S.D	Mean (%)	S.D	Mean (%)	S.D
	22,452	42.22	0.49	32.27	0.46	26.27	0.44
Economic quantile							
Poorest	3217	57.90	0.49	53.45	0.49	47.12	0.49
Poor	3500	56.63	0.49	45.02	0.49	37.91	0.48
Middle	3854	43.72	0.49	31.80	0.46	26.10	0.43
Rich	5090	37.37	0.48	25.68	0.43	19.38	0.39
Richest	6791	26.89	0.44	17.21	0.37	12.14	0.32
Sector							
Rural	11,867	47.20	0.49	37.31	0.48	31.39	0.46
Urban	10,585	34.22	0.47	24.16	0.42	18.04	0.38
Sex							
Male	16,368	43.96	0.49	33.80	0.47	27.46	0.44
Female	6082	38.30	0.48	28.82	0.45	23.58	0.42
Social group							
SC	3121	47.33	0.49	38.55	0.48	31.82	0.46
ST	1772	46.29	0.49	40.37	0.49	32.77	0.46
OBC	8552	44.13	0.49	32.85	0.46	26.45	0.44
Others	9007	37.58	0.48	28.04	0.44	22.93	0.42
Religion							
Hinduism	17,170	43.09	0.49	32.77	0.46	26.70	0.44
Islam	2717	41.57	0.49	32.93	0.46	27.04	0.44
Christian	1509	33.55	0.47	23.45	0.42	18.61	0.38
Others	1056	35.52	0.47	30.10	0.45	23.69	0.42
Insurance coverage							
Government	4214	37.67	0.48	26.83	0.44	22.93	0.42
Private	892	22.49	0.41	17.78	0.38	11.88	0.32
Others	134	30.18	0.45	25.21	0.43	17.72	0.38
None	17,212	44.46	0.49	34.47	0.47	27.90	0.44
Rank-weighted headcount (%)	22,452	53.95	0.63	43.16	0.62	35.85	0.60

Concentration Index headcount	Total obs.	Index value	s.e.*	Index value	s.e.*	Index value	s.e.*
	22,452	− 0.277	0.021*	− 0.337	0.021*	− 0.364	0.022*

*Source* Author's computation using NSSO 75th round, 2018.

*p-value < 0.01, *s.e.* standard error, *S.D.* standard deviation.

**Table 6:** Intensity of catastrophic health expenditure of elderly population (Tendulkar).

CHE as a share of household usual consumption expenditure (%)							
Threshold (%)	Total obs.	20%		30%		40%	
Overshoot (%)							
		Mean (%)	S.D	Mean (%)	S.D	Mean (%)	S.D
	22,452	57.8	4.17	54.12	4.16	51.21	4.15
Economic quantile							
Poorest	3217	228.58	9.88	222.99	9.87	218.00	9.86
Poor	3500	47.70	1.44	42.62	1.43	38.41	1.41
Middle	3854	26.73	0.99	23.07	0.98	20.21	0.96
Rich	5090	19.52	0.76	16.42	0.74	14.24	0.72
Richest	6791	11.12	0.49	8.97	0.47	7.50	0.46
Sector							
Rural	11,867	78.83	5.23	74.64	5.23	71.21	5.22
Urban	10,585	23.98	1.07	21.11	1.06	19.04	1.04
Sex							
Male	16,368	60.85	4.72	57.01	4.71	53.96	4.70
Female	6082	50.93	2.54	47.61	2.53	45.02	2.51
Social group							
SC	3121	110.61	5.32	106.27	5.30	102.78	5.29
ST	1772	59.16	4.30	54.86	4.29	51.08	4.28
OBC	8552	62.83	5.29	59.03	5.28	56.09	5.27
Others	9007	29.60	1.23	26.39	1.21	23.87	1.20
Religion							
Hinduism	17,170	59.48	4.37	55.73	4.36	52.77	4.35
Islam	2717	49.31	2.49	45.63	2.48	42.69	2.47
Christian	1509	22.43	0.80	19.61	0.78	17.48	0.76
Others	1056	91.55	5.95	88.33	5.94	85.70	5.93
Insurance coverage							
Government	4214	51.25	3.49	48.06	3.48	45.62	3.47
Private	892	15.59	2.47	13.69	2.46	12.25	2.46
Others	134	22.20	0.68	19.36	0.66	17.21	0.64
None	17,212	61.76	4.41	57.85	4.40	54.74	4.39
Rank-weighted overshoot (%)	22,452	90.95	6.57	86.55	6.66	82.91	6.73
Mean positive overshoot (%)	22,452	57.80	4.17	54.12	4.16	51.21	4.15

Concentration Index overshoot	Total obs.	Index value	s.e.*	Index value	s.e.*	Index value	s.e.*
	22,452	− 0.573	0.085*	− 0.599	0.091*	− 0.619	0.095*

*Source* Author's computation using NSSO 75th round, 2018.

*p-value < 0.01, *s.e.* standard error, *S.D.* standard deviation.

**Table 7:** Incidence of catastrophic health expenditure of elderly population (Rangarajan).

CHE as a share of household usual consumption expenditure (%)							
Threshold (%)	Total obs.	20%		30%		40%	
Headcount (%)							
		Mean (%)	S.D	Mean (%)	S.D	Mean (%)	S.D
	22,452	44.20	0.49	34.77	0.47	28.82	0.45
Economic quantile							
Poorest	3217	43.91	0.49	42.14	0.49	38.89	0.48
Poor	3500	63.95	0.48	53.10	0.49	46.74	0.49
Middle	3854	52.13	0.49	38.97	0.48	32.40	0.46
Rich	5090	41.02	0.49	31.01	0.46	22.89	0.42
Richest	6791	29.88	0.45	19.45	0.39	14.18	0.34
Sector							
Rural	11,867	48.28	0.49	38.66	0.48	33.26	0.47
Urban	10,585	37.64	0.48	28.5	0.45	21.68	0.41
Sex							
Male	16,368	45.87	0.49	36.16	0.48	30.19	0.45
Female	6082	40.44	0.49	31.62	0.46	25.72	0.43
Social group							
SC	3121	46.62	0.49	39.61	0.48	34.44	0.47
ST	1772	43.65	0.49	36.75	0.48	33.88	0.47
OBC	8552	45.44	0.49	36.08	0.48	29.25	0.45
Others	9007	41.94	0.49	31.10	0.46	25.36	0.43
Religion							
Hinduism	17,170	45.02	0.49	35.44	0.47	29.37	0.45
Islam	2717	43.30	0.49	34.05	0.47	28.75	0.45
Christian	1509	37.70	0.48	26.88	0.44	21.22	0.40
Others	1056	36.58	0.48	31.84	0.46	26.19	0.43
Insurance coverage							
Government	4214	39.80	0.48	30.20	0.45	23.34	0.42
Private	892	27.50	0.44	20.02	0.40	18.46	0.38
Others	134	33.35	0.47	24.25	0.42	16.70	0.37
None	17,212	46.24	0.49	36.76	0.48	30.85	0.46
Rank-weighted headcount (%)	22,452	53.01	0.59	43.94	0.60	37.63	0.59

Concentration Index headcount	Total obs.	Index value	s.e.*	Index value	s.e.*	Index value	s.e.*
	22,452	− 0.199	0.021*	− 0.263	0.022*	− 0.305	0.023*

*Source* Author's computation using NSSO 75th round, 2018.

*p-value < 0.01, *s.e.* standard error, *S.D.* standard deviation.

**Table 8:** Intensity of catastrophic health expenditure of elderly population (Rangarajan).

CHE as a share of household usual consumption expenditure (%)							
Threshold (%)	Total obs.	20%		30%		40%	
Overshoot (%)							
		Mean (%)	S.D	Mean (%)	S.D	Mean (%)	S.D
	22,452	92.19	8.78	88.27	8.77	85.10	8.76
Economic quantile							
Poorest	3217	361.24	20.91	356.94	20.90	352.88	20.89
Poor	3500	95.41	3.94	89.55	3.93	84.57	3.92
Middle	3854	41.08	1.59	36.51	1.58	32.98	1.56
Rich	5090	25.48	0.93	21.92	0.91	19.21	0.89
Richest	6791	12.91	0.53	10.51	0.52	8.85	0.50
Sector							
Rural	11,867	123.80	10.96	119.45	10.96	115.87	10.95
Urban	10,585	41.37	2.69	38.12	2.68	35.62	2.67
Sex							
Male	16,368	77.93	5.19	73.85	5.18	70.57	5.18
Female	6082	124.24	13.77	120.65	13.76	117.75	13.75
Social group							
SC	3121	191.65	18.17	187.35	18.17	183.62	18.16
ST	1772	130.21	7.40	126.27	7.39	122.77	7.38
OBC	8552	91.53	5.92	87.46	5.91	84.19	5.90
Others	9007	45.45	3.53	41.83	3.53	39.05	3.52
Religion							
Hinduism	17,170	79.95	5.20	75.94	5.19	72.71	5.18
Islam	2717	78.92	4.31	75.11	4.30	72.00	4.29
Christian	1509	32.84	1.91	29.61	1.90	27.27	1.89
Others	1056	487.53	38.41	484.14	38.40	481.2	38.39
Insurance coverage							
Government	4214	67.97	5.99	64.50	5.98	61.83	5.97
Private	892	15.71	0.55	13.51	0.53	11.60	0.51
Others	134	16.80	0.54	14.19	0.52	12.22	0.50
None	17,212	102.74	9.60	98.60	9.59	95.23	9.59
Rank-weighted overshoot (%)	22,452	147.50	14.05	143.01	14.21	139.23	14.34
Mean positive overshoot (%)	22,452	92.19	8.78	88.27	8.77	85.10	8.76

Concentration Index overshoot	Total obs.	Index value	s.e.*	Index value	s.e.*	Index value	s.e.*
	22,452	− 0.599	0.158*	− 0.620	0.165*	− 0.636	0.171*

*Source* Author's computation using NSSO 75th round, 2018.

*p-value < 0.01, *s.e.* standard error, *S.D.* standard deviation.

The weighted catastrophic headcount of the elderly population is greater than the unweighted and shows similar trends to the unweighted. It shows that the elderly population exceeds the various threshold levels and is inclined towards being poorer. For instance, after applying weight, 55.58% of the elderly population shows total OOPHS exceeding 10% of total household usual consumption expenditure; however, in the case of unweighted headcount, it is 46.51%. At the 40% threshold level, the weighted (unweighted) catastrophic headcount is 16.16% (13.71%). Similarly, for both Tendulkar and Rangarajan committee poverty line, the weighted catastrophic headcount is 35.85% and 37.63% at the 40% threshold level, higher than the unweighted catastrophic headcount.

**Figure 3:**
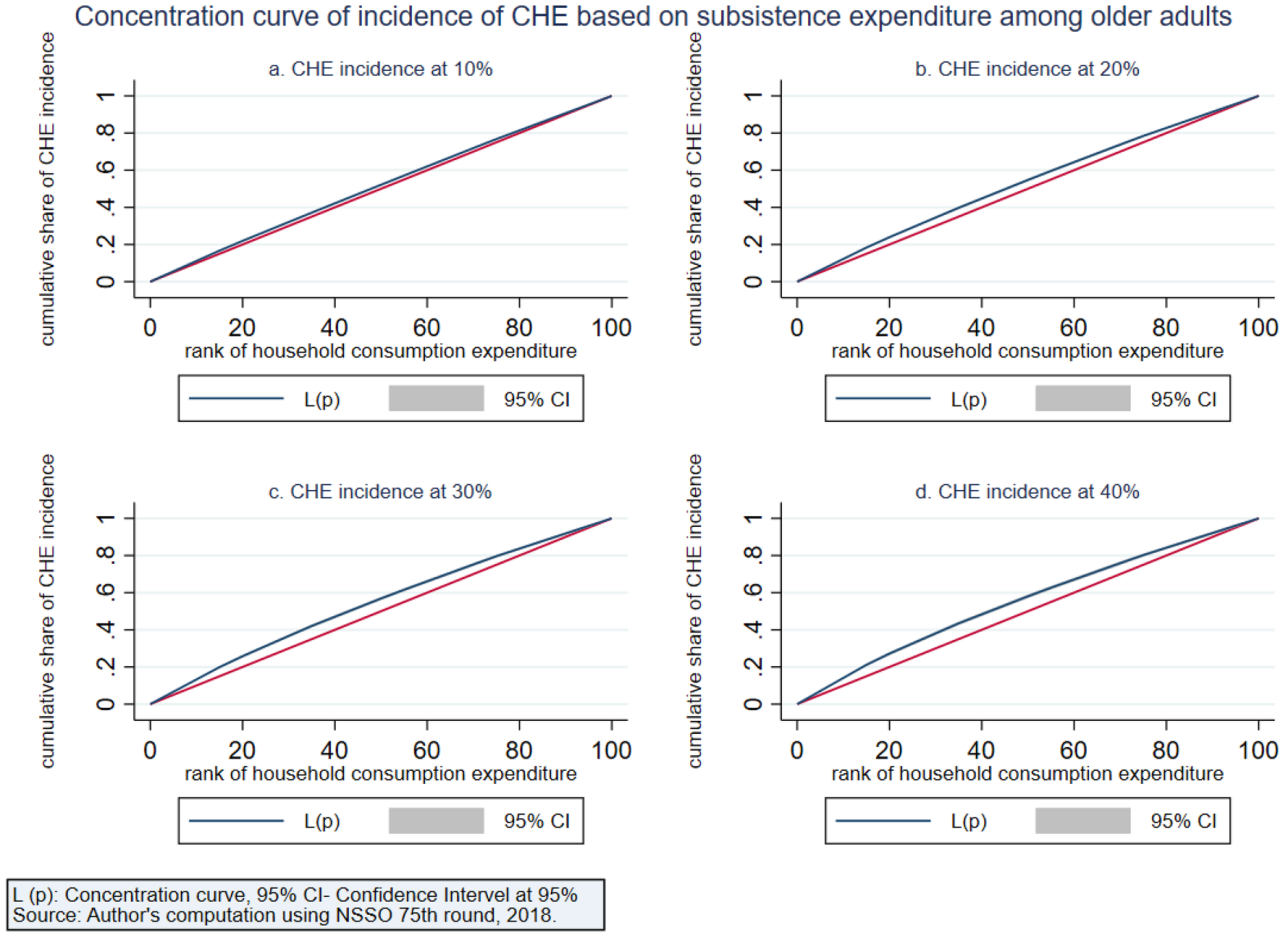
Concentration curve of incidence of CHE on subsistence expenditure among older adults.

**Figure 4:**
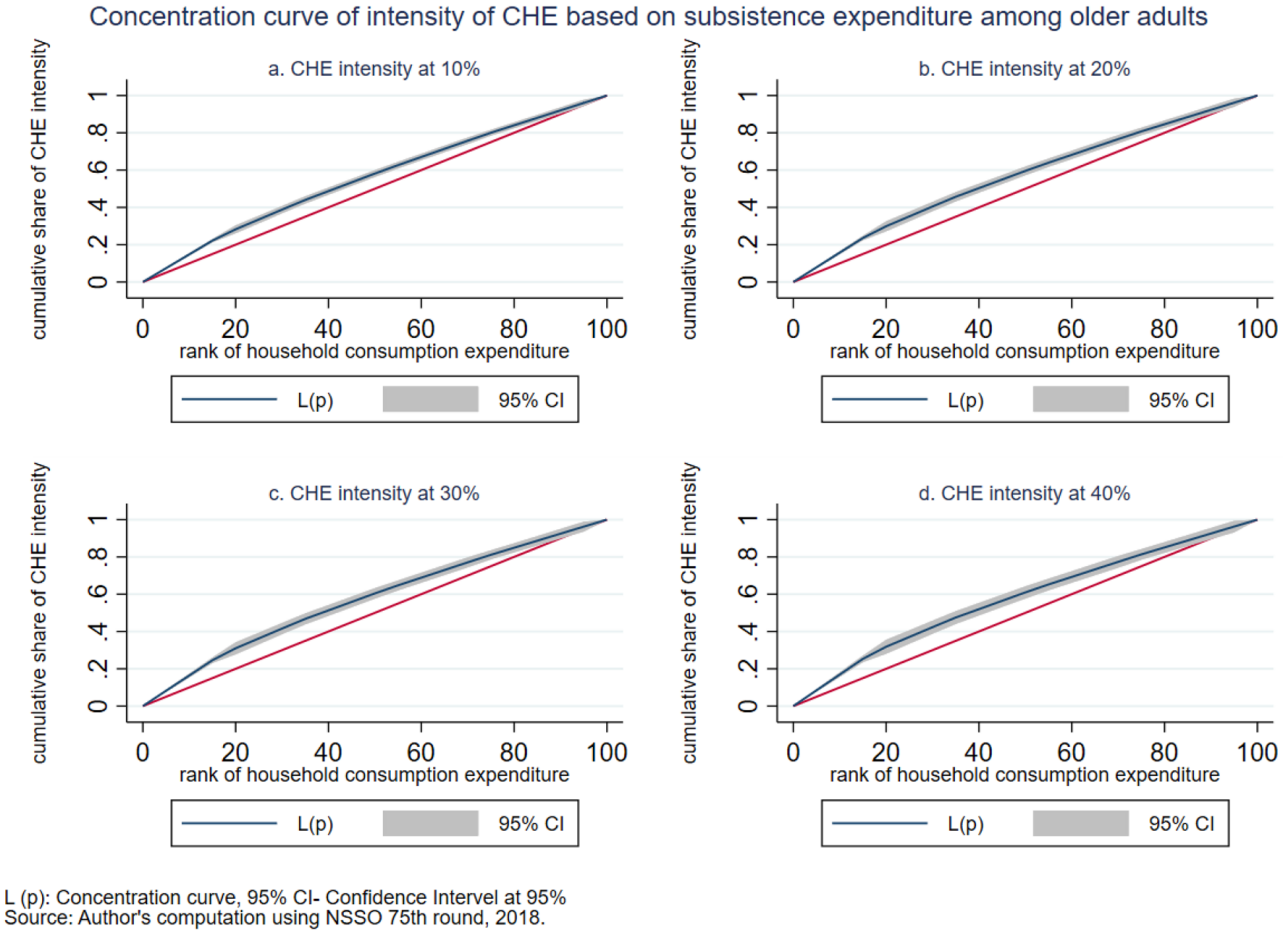
Concentration curve of intensity of CHE based on subsistence expenditure among older adults.

**Figure 5:**
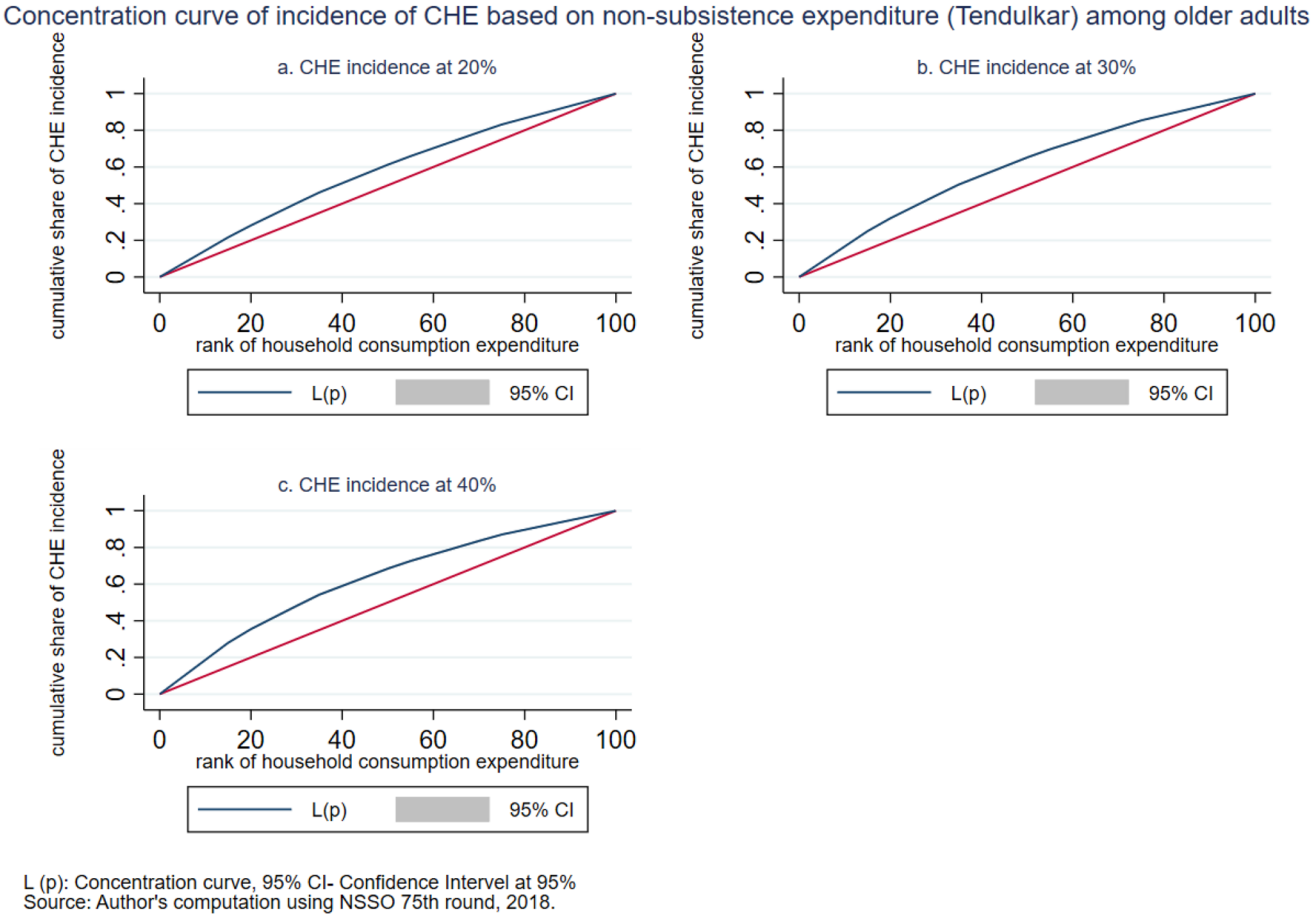
Concentration curve of incidence of CHE based on non-subsistence expenditure (Tendulkar) among older adults.

**Figure 6:**
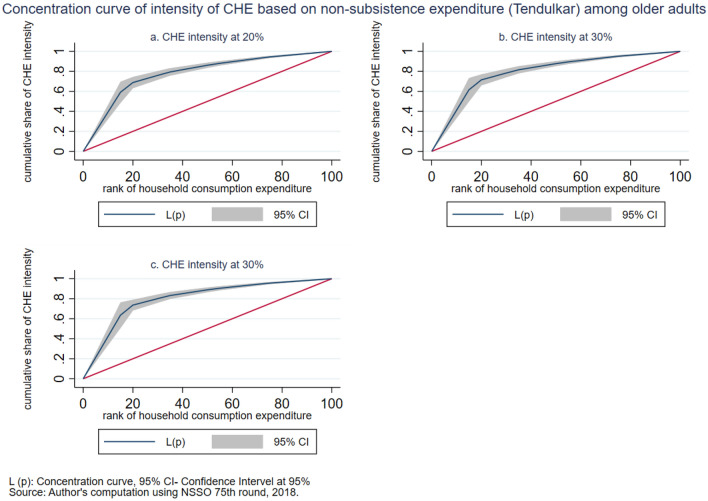
Concentration curve of intensity of CHE based on non-subsistence expenditure (Tendulkar) among older adults.

**Figure 7:**
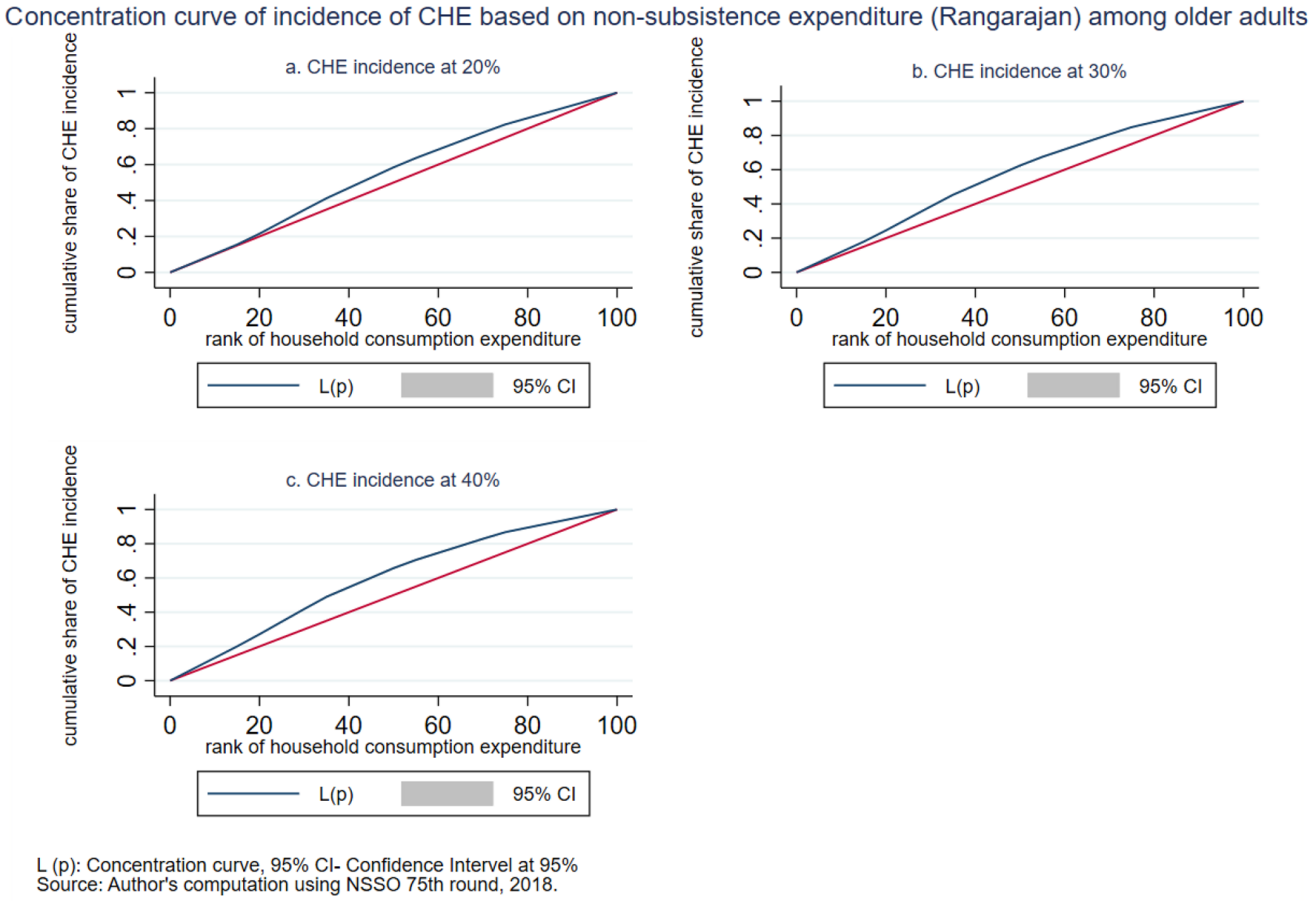
Concentration curve of incidence of CHE based on non-subsistence expenditure (Rangarajan) among older adults.

**Figure 8:**
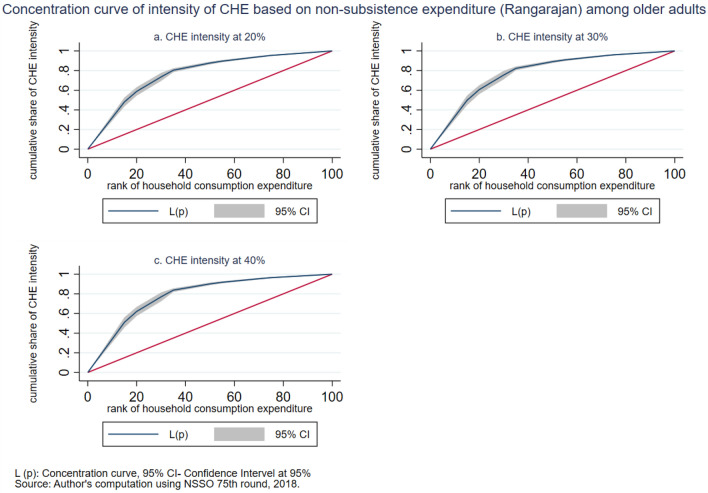
Concentration curve of intensity of CHE based on non-subsistence expenditure (Rangarajan) among older adults.

The intensity of CHE is measured by the overshoot method. The mean overshoot shows a decreasing trend if we increase the threshold level. The mean overshoot at 10% and 40% of total household usual consumption expenditure are 16.1% and 8.67%, respectively. Whereas in the case of non-subsistence spending, as per Tendulkar committee poverty line (Rangarajan committee poverty line), the mean overshoot is 57.8% (92.19%) at 20% and 51.21% (85.1%) at 40% threshold level. This result reports a substantially high overshoot which means that the elderly population exceeding the threshold level has a higher chance of moving toward poverty. The mean positive overshoot shows that the intensity of occurrence of CHE among the elderly population is high and increases over the threshold levels. All the concentration indices of headcount and overshoot are negative and significant (*p* < 0.001), which means that the concentration of catastrophic spending is higher among the poorest elderly population. The concentration curve in Figs. 3, 4, 5, 6, 7 and 8 show that the inequality of incidence and intensity of CHE persists among the poorest elderly population, and it increases with the threshold levels.

### Incidence of impoverishment

The poverty measurement before and after OOPHS is given in Table 9**,** and the sector-wise poverty measure is in Table 10. We used both Tendulkar and Rangarajan committee poverty lines to measure the incidence of impoverishment of the elderly population. By the extent of the Tendulkar (Rangarajan) committee poverty line, 4.32% (10.67%) of the elderly population live below the poverty line before accounting for any healthcare spending. After considering healthcare spending, the poverty headcount rises to 14.21% (22.21%) by the Tendulkar (Rangarajan) committee poverty line measure. This result shows a substantial increase in poverty measures, which accounted for 9.89% (11.54%) of the elderly population. The average poverty gap deficit to achieve the poverty line is INR 97.7 (INR 120.04) per month, according to Tendulkar (Rangarajan) committee. The average normalized poverty gap of the elderly population is 56.45% (57.91%). This means that the elderly population has a deficit of income or consumption among those who are already below the poverty line and will be further pushed into the depth of poverty. In Table 9, it is shown that rural pre- and post-poverty is higher than urban counterparts for both Tendulkar as well as Rangarajan poverty line. The poverty gap deficit to achieve the Tendulkar (Rangarajan) poverty line in rural and urban areas are INR 101.74 (INR 121.45) and INR 88.57 (INR 114.98) per month. The depth of poverty is higher in rural areas compared to urban areas.

**Table 9:** Poverty measurement before and after out-of-pocket health spending.

	Tendulkar	Rangarajan
Poverty headcount (H)		
Pre-H	4.32%	10.67%
Post-H	14.21%	22.21%
H = pre-H – post-H	9.89%	11.54%
Poverty gap (G)		
Pre-G	6.64	23.27
Post-G	104.34	143.31
G = pre-G – post-G	97.70	120.04
Normalized poverty gap (NG)		
Pre-NG	7.09%	18.99%
Post-NG	63.54%	76.90%
NG = pre-NG – post-NG	56.45%	57.91%

*Source* Author's computation using NSSO 75th round, 2018.

**Table 10:** Sector-wise poverty measurement before and after out-of-pocket health spending.

	Tendulkar		Rangarajan	
	Rural	Urban	Rural	Urban
Poverty line				
Pre-PL	816	1000	972	1407
Post-PL	816	1000	972	1407
Poverty headcount (H)				
Pre-H	5.06%	2.94%	11.96%	8.20%
Post-H	17.47%	8.25%	25.76%	15.56%
H = pre-H – post-H	12.41%	5.31%	13.80%	7.36%
Poverty gap (G)				
Pre-G	7.33	5.29	20.87	26.93
Post-G	109.07	93.86	142.32	141.91
G = pre-G – post-G	101.74	88.57	121.45	114.98
Normalized poverty gap (NG)				
Pre-NG	8.38%	4.68%	19.11%	18.37%
Post-NG	73.62%	44.68%	86.88%	57.92%
NG = pre-NG – post-NG	65.24%	40.00%	67.77%	39.55%

*Source* Author's computation using NSSO 75th round, 2018.

## Discussion

The outcome of this paper presents a significant inequality of OOPHS on elderly health at the levels of economic quantile, sector, gender, social group, and religion. The most affluent population is more likely to pay more for healthcare services than the poorest. It shows that the ability to pay for healthcare services is concentrated among the richest population, which is also confirmed through the concentration indices, reflecting that the accessibility and affordability of healthcare services are costly. Previous literature suggests that OOPHS among the most affluent population is high, and low-income people are less likely to spend on healthcare services due to high costs or just ignore the illness to take care of^32,33^. This result might imply that the elderly population pays a large amount of out-of-pocket due to the direct relationship between aging and health deterioration. At old age, ignorance of healthcare would be likely to compromise their quality of life. The poor older adults might not be able to afford the increasing cost of healthcare services, and therefore their quality of life might worsen.

Above mention Fig. 2 shows that Indian elderly spends, on average, approximately 17% of their consumption budget on healthcare services. For both inpatient (17%) and outpatient (14%) services, OOPHS is relatively high in the case of the elderly population. But the share of out-of-pocket consumption budget is quite high for both inpatient and outpatient services among the poorest compared to the richest. The poorest population spends twice as much of their consumption budget on healthcare services than the richest counterpart. This result is consistent with previous literatures that the increased share of OOPHS scenario among the poorest compared to the richest is observed as a very regressive in low-income countries^34–38^. Higher spending on healthcare might occur due to income gap across economic quantiles and less expensive but frequent outpatient visits than hospitalization might increase their OOPHS. Older people need healthcare support and cannot be ignored these services at old age which can reflect the financial burden on the bread-winner due to high OOPHS.

There is no well-defined threshold level to measure the incidence of CHE^4^. Hence, it is better to take a range of threshold levels for both subsistence and non-subsistence expenditure methods to estimate the incidence of CHE. The above result, Tables 3, 4, 5, 6, 7 and 8, shows that 46.5% and 13.7% of the elderly population incurred CHE that exceeded 10% and 40% threshold levels based on subsistence expenditure, whereas 42.2% (26.2%) and 26.2% (28.8%) of the elderly population incurred CHE that exceeded 20% and 40% of non-subsistence expenditure according to Tendulkar (Rangarajan) committee poverty line. Past studies found, mainly based on at 40% threshold level of non-subsistence expenditure, 7% of CHE incidence among older adults^23^ and recently, 19% of CHE incidence among households with an older adults^39^. Similarly, In China, the CHE, measured at 40% of non-subsistence expenditure, among elderly has been increased from 12.9% in 2011 to 27.9% in 2015^40^. The overall incidence of CHE for health services in India was 12.5% (2004), 13.4% (2014), and 9.1% (2018) at the 40% threshold level^41^. Finding in this paper is solely among elderly population with subsistence expenditure and two different measurement of non-subsistence expenditure. The occurrence of CHE among the elderly population by non-subsistence expenditure measures is much higher than in subsistence expenditure measures. This estimate reflects that a large amount of the Indian population spends on essential consumption^42^, which is seen through the past literature that people in low-income countries spend primarily on food and other necessities^43^.

As above mention result, Tables 3, 4, 5, 6, 7 and 8, shows that socio-economic inequality persists in accessing and affordability of healthcare services between rich-poor, rural–urban, male–female, social groups, and religions. The incidence and intensity of CHE among the elderly population being poor, living in rural areas, being a male, belongs from schedule castes, and no insurance coverage is higher than their respective counterparts. This result is consistent with past studies which show that these variables are the risk factors of incurring CHE^4,11,13^. The intensity of CHE among older adults is quite high in non-subsistence expenditure methods than the subsistence one. Within non-subsistence expenditure methods, Rangarajan method of non-subsistence expenditure shows an extreme intensity of facing a substantial risk of CHE among older adults due to OOPHS. Low income, lack of economic independence, expensive healthcare services, low coverage of health insurance, frequent visits of quackery, and private medical motive of profit maximization, lack of healthcare facilities, long distance of medical care from remote areas, transportation facilities at midnight and its costs, and patriarch lineage of property rights can be some of the reasons of facing the risk of CHE among older adults. Overall, at a 10% threshold level of subsistence expenditure or a 20% threshold level of non-subsistence expenditure, every socio-economic group bears the high expenses for elderly healthcare services. As we increase the threshold up to 40% for both subsistence and non-subsistence expenditure, the occurrence of CHE declines, which means that marginalized socio-economic groups either they cannot bear more expenses for elderly healthcare services or not seeking healthcare services at all.

Further, we measure the incidence of impoverishment due to CHE. We utilize both Tendulkar (2011–12) as well as Rangarajan (2014) committee poverty lines to estimate the incidence of poverty among the elderly population in India. In Table 9, the incidence of poverty headcount of the elderly population before accounting for the healthcare payments is 4.3% (10.6%) by Tendulkar (Rangarajan) approach, and after considering healthcare payments, the poverty headcount increases to 14.2% (22.2%) respectively. 9.8% (11.5%) of the elderly population are being pushed towards poverty after OOPHS. It shows that more than half of the elderly population below the poverty line are further pushed into extreme poverty, which might be a poverty trap for them. According to the World Bank poverty estimate (a person living on less than 1.90 US dollars a day), about 20% of India's population lives in extreme poverty (World Bank, 2017). According to the NITI Aayog report (2021), the overall multidimensional headcount ratio is 25.01%, in which rural and urban areas consist of 32.75% and 8.81%, respectively. In Table 10, the incidence of poverty headcount in rural areas is greater than the urban areas. According to Tendulkar (Rangarajan) committee, 12.4% (13.8%) and 5.3% (7.3%) of the elderly population are falling into poverty due to healthcare payments in rural and urban areas, respectively. The rural elderly population below the poverty line is further pushed into deep poverty than their urban counterparts. If we interpret these results in numbers, that would be quite high since India is the second most populous country in the world, where 104 million consists of the elderly population^44^, and still rising. Moreover, it is a suggestion for health policy makers to implement financial protection in such a way to minimize the rural–urban disparities among older adults in India. Policy makers and stakeholders in the field of healthcare should think about proper financial protection along with vision to improve the quality of life and longevity among older adults due to demographic shifts towards rapidly growing ageing population In India as well as globally.

### Limitations

Some limitations are observed too during this study. First, all the findings are based on cross-sectional survey data. A panel data approach would be more fruitful in capturing the incidence and intensity of CHE and impoverishment among the elderly population. Second, for estimating overall OOPHS, including both inpatient and outpatient costs, the outpatient cost is given for 15 days recall period, which we transform into the reference of the inpatient cost, which is a 365-day recall period. However, the results are suitable since we estimated the inpatient (365 days recall period) and outpatient (15 days recall period) OOPHS and CHE separately (see Table 1 and Appendix 1 & Appendix 2); the outcomes are likely to follow a similar pattern. Third, we have not considered the loss of household income due to healthcare services. Fourth, the data do not capture those poor individuals who do not seek treatments because of various socio-economic barriers; therefore, this could lead to an underestimation of the incidence and intensity of CHE and impoverishment among the elderly population. Finally, poverty measures show a difference between rural and urban areas. Most of the elderly population who were pushed into poverty traps may belong to rural areas and urban slums.

## Conclusions

The findings reflect that the financial burden of OOPHS among the elderly population is substantially high in India. Financial protection is very much needed for the elderly population due to high OOPHS on healthcare services, and with aging, it is unavoidable not to seek healthcare facilities for the elderly population. Older adults belonging from low socio-economic backgrounds either spend a high share of their consumption budget on these services and forgo their consumption or do not seek treatment. Financial protection should be proportionate to population size to take care of, like those elderly population who are more likely to become impoverished due to healthcare payments. 81% of the elderly population does not have any health insurance (NSSO, 2018), and it has its limitations in terms and services, which further reduces the interest of insurers. Even those with health insurance did not mitigate their CHE^45^. Without proper financial support the healthcare requirements at old age are very crucial for improvement in quality of life but availing these services might result in high OOPHS which may result in CHE and further impoverishment among older adults in India. Therefore, there is an urgent need to consider alternative healthcare financing mechanisms (such as effective health insurance policy, investment in physical healthcare infrastructure and technology, human resources, and equally accessible healthcare resources at affordable prices to all) to take care of the burden of financial risk and will protect the elderly population from healthcare related impoverishment. That will also support the government’s goal of universal health coverage by 2030 (National Institution for Transforming India (NITI) Aayog, Government of India). The future direction of research could be the program evaluation regarding the implementation of financial support and health insurance schemes in reducing the risk of CHE among older adults.

### Supplementary Information

Supplementary Information 1.

Supplementary Information 2.

## Data Availability

The secondary datasets used and/or analyzed during the current study are available and can be access with valid registration from the public (Ministry of Statistics & Programme Implementation, Government of India, 2018) repository, (https://microdata.gov.in/nada43/index.php/catalog/152).

## References

[1] Goals, S.D. World Health Statistics (2022).

[2] MoHFW G. National Health Accounts: Estimates For India 2019–20. (2023). https://main.mohfw.gov.in/sites/default/files/5NHA_19-20_dt%2019%20April%202023_web_version_1.pdf

[3] Berki SE (1986). A look at catastrophic medical expenses and the poor. Health Aff..

[4] Azzani M, Roslani AC, Su TT (2019). Determinants of household catastrophic health expenditure: A systematic review. Malaysian J. Med. Sci..

[5] Kastor A, Mohanty SK (2018). Disease-specific out-of-pocket and catastrophic health expenditure on hospitalization in India: Do Indian households face distress health financing?. PLoS One.

[6] Liu S, Coyte PC, Fu M, Zhang Q (2021). Measurement and determinants of catastrophic health expenditure among elderly households in China using longitudinal data from the CHARLS. Int. J. Equity Health.

[7] Stauder J (2019). Unemployment, unemployment duration, and health: selection or causation?. Eur. J. Health Econ..

[8] Arsenijevic J, Pavlova M, Rechel B, Groot W (2016). Catastrophic health care expenditure among older people with chronic diseases in 15 European countries. PLoS One.

[9] Yadav J, Menon GR, John D (2021). Disease-specific out-of-pocket payments, catastrophic health expenditure and impoverishment effects in India: An analysis of national health survey data. Appl. Health Econ. Health Policy.

[10] Xu K, Evans DB, Kadama P (2006). Understanding the impact of eliminating user fees: Utilization and catastrophic health expenditures in Uganda. Soc. Sci. Med..

[11] Yardim MS, Cilingiroglu N, Yardim N (2010). Catastrophic health expenditure and impoverishment in Turkey. Health Policy (New York).

[12] Li Y, Wu Q, Xu L (2012). Factors affecting catastrophic health expenditure and impoverishment from medical expenses in China: Policy implications of universal health insurance. Bull. World Health Organ..

[13] Pal R (2012). Measuring incidence of catastrophic out-of-pocket health expenditure: With application to India. Int. J. Health Care Finance Econ..

[14] Grundy E (2006). Ageing and vulnerable elderly people : European Ageing and vulnerable elderly people: European perspectives. Ageing Soc..

[15] Guégan J (2018). Sustainable Development Goal #3, “health and well-being”, and the need for more integrative thinking. Vet México OA.

[16] Obermann K, Jowett MR, Alcantara MOO, Banzon EP, Bodart C (2006). Social health insurance in a developing country: The case of the Philippines. Soc. Sci. Med..

[17] Kapoor M, Nidhi Kaur K, Saeed S, Shannawaz M, Chandra A (2023). Impact of COVID-19 on healthcare system in India: A systematic review. J. Public health Res..

[18] Villarin, T. Social inclusion in the time of COVID-19 pandemic : Philippines Social inclusion in the time of COVID-19 pandemic (2020). 10.13140/RG.2.2.28270.84807

[19] Bose M, Dutta A (2015). Inequity in hospitalization care: A study on utilization of healthcare services in West Bengal, India. Int. J. Health Policy Manag..

[20] Baeten, R., Spasova, S. & Vanhercke, B. (2018) Inequalities in Access to Healthcare A Study of National Policies

[21] Srivastava S, Chauhan S, Patel R (2021). Socio-economic inequalities in the prevalence of poor self-rated health among older adults in India from 2004 to 2014: A decomposition analysis. Ageing Int..

[22] Ghosh S (2014). Trends and differentials in health care utilization pattern in India. J. Health Manag..

[23] Brinda EM, Kowal P, Attermann J, Enemark U (2015). Health service use, out-of-pocket payments and catastrophic health expenditure among older people in India: The WHO study on global AGEing and adult health (SAGE). J. Epidemiol. Community Health.

[24] Pandey P, Singh S (2017). Catastrophic health expenditure among geriatric population of Lucknow district, India. J. Geriatr. Ment. Health.

[25] Razzaque A, Id S, Ali SMZ (2022). Out-of-pocket payment for healthcare among urban citizens in Dhaka, Bangladesh. PLoS One.

[26] NSSO. Health in India: NSS 75th Round. Gov Rep. Published online 2020. http://mospi.nic.in/sites/default/files/publication_reports/NSS Report no. 586 Health in India.pdf

[27] WHO. Distribution of health payments and catastrophic expenditures Methodology DISCUSSION PAPER. *World Heal Organ Geneva*. Published online 2005. https://iris.who.int/bitstream/handle/10665/69030/EIP_?sequence=1

[28] Xu K, Evans DB, Kawabata K, Zeramdini R, Klavus J, Murray CJL (2003). Household catastrophic health expenditure: A multicountry analysis. Lancet.

[29] Van Doorslaer E (2007). Catastrophic payments for health care in Asia. Health Econ..

[30] Planning Commission of India. Press Note on Poverty Estimates, 2011–12 Government of India Planning Commission July 2013. *Press Inf Bur*. 1–10 (2013).

[31] Report R, The P, Group E. Press Information Bureau Government of India Planning Commission. *Press Inf Bur*. Published online 2019:25–27.

[32] Jane C, Thomas M (2012). Catastrophic health care spending and impoverishment in Kenya. BMC Health Serv. Res..

[33] Pandey A, Ploubidis GB, Clarke L, Dandona L (2018). Trends in catastrophic health expenditure in india: 1993 to 2014. Bull. World Health Organ..

[34] Van Damme W, Van Leemput L, Por I, Hardeman W, Meessen B (2004). Out-of-pocket health expenditure and debt in poor households: Evidence from Cambodia. Trop. Med. Int. Health.

[35] Ruger JP, Kim HJ (2007). Out-of-pocket healthcare spending by the poor and chronically III in the republic of Korea. Am. J. Public Health.

[36] Chaudhuri A, Roy K (2008). Changes in out-of-pocket payments for healthcare in Vietnam and its impact on equity in payments, 1992–2002. Health Policy (New York)..

[37] Sirag A, Nor NM (2021). Out-of-pocket health expenditure and poverty: Evidence from a dynamic panel threshold analysis. Healthcare.

[38] Wagstaff A, Eozenou P, Smitz M (2020). Out-of-pocket expenditures on health: A global stocktake. World Bank Res. Obs..

[39] Panda BK, Mohanty SK (2022). Catastrophic health spending among older adults in India: Role of multiple deprivation. Aging Health Res..

[40] Zhou Y, Wushouer H, Vuillermin D, Guan X, Shi L (2021). Does the universal medical insurance system reduce catastrophic health expenditure among middle-aged and elderly households in China? A longitudinal analysis. Eur. J. Health Econ..

[41] Mohanty SK, Dwivedi LK (2021). Addressing data and methodological limitations in estimating catastrophic health spending and impoverishment in India, 2004–18. Int. J. Equity Health.

[42] Panikkassery AS (2020). Impact of out of pocket health expenditure on consumption pattern of below poverty line households in India. Millenn Asia.

[43] Banerjee AV, Duflo E (2007). The economic lives of the poor. J. Econ. Perspect..

[44] Census of India 2011. Population Projections for India and States 2011–2036-Report of The Technical Group On Population Projections, July, 2020. *Gov India Rep*. Published online 2020:26–32. https://main.mohfw.gov.in/sites/default/files/Population_Projection_Report_2011-2036-upload_compressed_0.pdf

[45] Wang J, Zhu H, Liu H (2020). Can the reform of integrating health insurance reduce inequity in catastrophic health expenditure? Evidence from China. Int. J. Equity Health.

