# Supplementary material for: Incidence and intensity of catastrophic health expenditure and impoverishment among the elderly: an empirical evidence from India
Source: Sci Rep. 2024 Jul 10;14:15908. doi: 10.1038/s41598-024-55142-1 (PMC11237111; doi:10.1038/s41598-024-55142-1)
Supplement: Supplementary file 1 — Supplementary Information 1. [file 41598_2024_55142_MOESM1_ESM.docx]

**Appendix 1:** Supplementary tables

**Tables 11 and 12 report the incidence and intensity of CHE at subsistence expenditure due to inpatient services respectively.**

| **Table 11: Inpatient incidence of catastrophic health expenditure of elderly population** | | | | | | | | | | |
| --- | --- | --- | --- | --- | --- | --- | --- | --- | --- | --- |
| **CHE as a share of household usual consumption expenditure (%)** | | | | | | | | | | |
| **Threshold (%)** | | | **10%** | | **20%** | | **30%** | | **40%** | |
| **Headcount (%)** | | | | | | | | | | |
|  |  | **Total obs.** | **Mean (%)** | **S.D.** | **Mean (%)** | **S.D.** | **Mean (%)** | **S.D.** | **Mean (%)** | **S.D.** |
|  |  | 18,901 | 38.33 | 0.48 | 23.08 | 0.42 | 15.09 | 0.35 | 10.83 | 0.31 |
| **Economic quantile** | | | | | | | | | | |
| Poorest | | 2,832 | 40.35 | 0.49 | 25.94 | 0.43 | 18.10 | 0.38 | 12.22 | 0.32 |
| Poor | | 3,030 | 36.33 | 0.48 | 23.58 | 0.42 | 15.03 | 0.36 | 10.46 | 0.31 |
| Middle | | 3,338 | 37.27 | 0.48 | 22.92 | 0.42 | 14.71 | 0.35 | 10.32 | 0.30 |
| Rich | | 4,306 | 37.62 | 0.48 | 20.81 | 0.40 | 13.88 | 0.34 | 10.94 | 0.31 |
| Richest | | 5,395 | 39.67 | 0.49 | 22.58 | 0.41 | 14.18 | 0.35 | 10.34 | 0.30 |
| **Sector** | | | | | | | | | | |
| Rural | | 10,146 | 38.58 | 0.49 | 23.81 | 0.42 | 15.74 | 0.36 | 11.17 | 0.31 |
| Urban | | 8,755 | 37.89 | 0.48 | 21.78 | 0.41 | 13.94 | 0.34 | 10.23 | 0.30 |
| **Sex** | | | | | | | | | | |
| Male | | 14,765 | 38.27 | 0.49 | 22.75 | 0.41 | 14.99 | 0.35 | 10.96 | 0.31 |
| Female | | 4,134 | 38.55 | 0.49 | 24.22 | 0.42 | 15.45 | 0.36 | 10.40 | 0.30 |
| **Social group** | | | | | | | | | | |
| SC | | 2,664 | 33.85 | 0.47 | 19.08 | 0.39 | 10.61 | 0.30 | 7.09 | 0.25 |
| ST | | 1,593 | 29.27 | 0.45 | 17.06 | 0.37 | 11.02 | 0.31 | 6.40 | 0.24 |
| OBC | | 7,314 | 39.18 | 0.49 | 23.87 | 0.42 | 15.32 | 0.36 | 10.91 | 0.31 |
| Others | | 7,330 | 40.43 | 0.49 | 24.62 | 0.43 | 17.25 | 0.37 | 12.89 | 0.33 |
| **Religion** | | | | | | | | | | |
| Hinduism | | 14,505 | 39.18 | 0.49 | 24.01 | 0.42 | 15.81 | 0.36 | 11.34 | 0.32 |
| Islam | | 2,246 | 30.78 | 0.46 | 16.45 | 0.37 | 10.41 | 0.30 | 7.95 | 0.27 |
| Christian | | 1,265 | 45.05 | 0.50 | 26.72 | 0.44 | 17.45 | 0.38 | 11.14 | 0.31 |
| Others | | 885 | 38.21 | 0.48 | 21.78 | 0.41 | 13.11 | 0.34 | 9.40 | 0.29 |
| **Insurance coverage** | | | | | | | | | | |
| Government | | 3,486 | 32.51 | 0.47 | 19.61 | 0.39 | 12.34 | 0.33 | 9.30 | 0.29 |
| Private | | 708 | 28.67 | 0.45 | 12.47 | 0.33 | 8.77 | 0.28 | 6.26 | 0.24 |
| Others | | 118 | 26.65 | 0.44 | 16.89 | 0.37 | 11.88 | 0.32 | 11.81 | 0.32 |
| None | | 14,589 | 40.28 | 0.49 | 24.45 | 0.42 | 16.08 | 0.36 | 11.41 | 0.31 |
| **Rank-weighted headcount (%)** | | 18,901 | 38.21 | 0.48 | 23.99 | 0.43 | 15.90 | 0.38 | 11.12 | 0.32 |
| **Concentration Index headcount** | | **Total obs.** | **Index value** | **s.e.*** | **Index value** | **s.e.*** | **Index value** | **s.e.*** | **Index value** | **s.e.*** |
|  | | 18,901 | 0.003 | 0.017 | -0.039 | 0.021* | -0.053 | 0.023** | -0.026 | 0.027 |
| **Source:** Author's computation using NSSO 75th round, 2018. | | | | | | | | | | |
| *p-value<0.1, **p-value<0.05, s.e.- standard error, S.D.- standard deviation | | | | | | | | | | |

| **Table 12: Inpatient intensity of catastrophic health expenditure of elderly population** | | | | | | | | | | |
| --- | --- | --- | --- | --- | --- | --- | --- | --- | --- | --- |
| **CHE as a share of household usual consumption expenditure (%)** | | | | | | | | | | |
| **Threshold (%)** | | | **10%** | | **20%** | | **30%** | | **40%** | |
| **Overshoot (%)** | | | | | | | | | | |
|  |  | **Total obs.** | **Mean (%)** | **S.D.** | **Mean (%)** | **S.D.** | **Mean (%)** | **S.D.** | **Mean (%)** | **S.D.** |
|  |  | 18,901 | 12.80 | 0.45 | 9.83 | 0.43 | 7.96 | 0.41 | 6.67 | 0.40 |
| **Economic quantile** | | | | | | | | | | |
| Poorest | | 2,832 | 14.30 | 0.45 | 11.11 | 0.43 | 8.94 | 0.41 | 7.42 | 0.39 |
| Poor | | 3,030 | 12.63 | 0.42 | 9.70 | 0.40 | 7.80 | 0.38 | 6.55 | 0.37 |
| Middle | | 3,338 | 12.34 | 0.39 | 9.39 | 0.37 | 7.55 | 0.35 | 6.28 | 0.33 |
| Rich | | 4,306 | 11.95 | 0.44 | 9.14 | 0.42 | 7.43 | 0.40 | 6.20 | 0.39 |
| Richest | | 5,395 | 12.87 | 0.51 | 9.87 | 0.49 | 8.10 | 0.48 | 6.89 | 0.46 |
| **Sector** | | | | | | | | | | |
| Rural | | 10,146 | 12.93 | 0.40 | 9.88 | 0.38 | 7.93 | 0.36 | 6.58 | 0.34 |
| Urban | | 8,755 | 12.57 | 0.52 | 9.73 | 0.50 | 8.02 | 0.49 | 6.82 | 0.47 |
| **Sex** | | | | | | | | | | |
| Male | | 14,765 | 12.70 | 0.44 | 9.74 | 0.42 | 7.89 | 0.40 | 6.60 | 0.39 |
| Female | | 4,134 | 13.17 | 0.47 | 10.14 | 0.45 | 8.21 | 0.44 | 6.90 | 0.42 |
| **Social group** | | | | | | | | | | |
| SC | | 2,664 | 8.87 | 0.30 | 6.30 | 0.28 | 4.82 | 0.26 | 3.94 | 0.25 |
| ST | | 1,593 | 6.85 | 0.22 | 4.65 | 0.20 | 3.29 | 0.18 | 2.41 | 0.17 |
| OBC | | 7,314 | 13.04 | 0.42 | 10.00 | 0.40 | 8.10 | 0.39 | 6.80 | 0.37 |
| Others | | 7,330 | 14.96 | 0.54 | 11.79 | 0.52 | 9.73 | 0.50 | 8.23 | 0.49 |
| **Religion** | | | | | | | | | | |
| Hinduism | | 14,505 | 13.57 | 0.47 | 10.50 | 0.45 | 8.55 | 0.43 | 7.20 | 0.42 |
| Islam | | 2,246 | 8.02 | 0.27 | 5.78 | 0.25 | 4.45 | 0.23 | 3.55 | 0.22 |
| Christian | | 1,265 | 14.26 | 0.47 | 10.80 | 0.45 | 8.65 | 0.44 | 7.26 | 0.42 |
| Others | | 885 | 11.18 | 0.43 | 8.28 | 0.41 | 6.63 | 0.40 | 5.57 | 0.38 |
| **Insurance coverage** | | | | | | | | | | |
| Government | | 3,486 | 9.56 | 0.29 | 7.08 | 0.27 | 5.54 | 0.25 | 4.47 | 0.23 |
| Private | | 708 | 7.98 | 0.30 | 5.94 | 0.28 | 4.88 | 0.27 | 4.12 | 0.25 |
| Others | | 118 | 10.15 | 0.32 | 8.05 | 0.30 | 6.44 | 0.28 | 5.26 | 0.26 |
| None | | 14,589 | 13.84 | 0.48 | 10.70 | 0.46 | 8.71 | 0.45 | 7.34 | 0.43 |
| **Rank-weighted overshoot (%)** | | 18,901 | 13.05 | 0.45 | 10.04 | 0.44 | 8.09 | 0.42 | 6.76 | 0.41 |
| **Mean positive overshoot (%)** | | 18,901 | 12.80 | 0.45 | 9.83 | 0.43 | 7.96 | 0.41 | 6.67 | 0.40 |
| **Concentration Index overshoot** | | **Total obs.** | **Index value** | **s.e.*** | **Index value** | **s.e.*** | **Index value** | **s.e.*** | **Index value** | **s.e.*** |
|  | | 18,901 | -0.019 | 0.023 | -0.021 | 0.028 | -0.017 | 0.033 | -0.012 | 0.037 |
| **Source:** Author's computation using NSSO 75th round, 2018. | | | | | | | | | | |
| *p-value<0.01, s.e.- standard error, S.D.- standard deviation | | | | | | | | | | |

**Tables 13 and 14 report the incidence and intensity of CHE at non-subsistence expenditure (Tendulkar committee poverty line) due to inpatient services respectively.**

| **Table 13: Inpatient incidence of catastrophic health expenditure of elderly population (Tendulkar)** | | | | | | | | |
| --- | --- | --- | --- | --- | --- | --- | --- | --- |
| **CHE as a share of household usual consumption expenditure (%)** | | | | | | | | |
| **Threshold (%)** | | | **20%** | | **30%** | | **40%** | |
| **Headcount (%)** | | | | | | | | |
|  |  | **Total obs.** | **Mean (%)** | **S.D.** | **Mean (%)** | **S.D.** | **Mean (%)** | **S.D.** |
|  |  | 18,901 | 36.44 | 0.48 | 27.55 | 0.44 | 22.35 | 0.41 |
| **Economic quantile** | | | | | | | | |
| Poorest | | 2,832 | 51.24 | 0.50 | 42.01 | 0.49 | 37.93 | 0.48 |
| Poor | | 3,030 | 39.96 | 0.49 | 32.49 | 0.47 | 26.92 | 0.44 |
| Middle | | 3,338 | 35.32 | 0.48 | 27.56 | 0.44 | 20.69 | 0.40 |
| Rich | | 4,306 | 31.26 | 0.46 | 21.24 | 0.41 | 16.52 | 0.37 |
| Richest | | 5,395 | 27.94 | 0.45 | 18.36 | 0.39 | 13.45 | 0.34 |
| **Sector** | | | | | | | | |
| Rural | | 10,146 | 38.26 | 0.48 | 29.69 | 0.45 | 24.41 | 0.43 |
| Urban | | 8,755 | 33.22 | 0.47 | 23.75 | 0.42 | 18.68 | 0.39 |
| **Sex** | | | | | | | | |
| Male | | 14,765 | 36.60 | 0.48 | 27.78 | 0.45 | 22.50 | 0.42 |
| Female | | 4,134 | 35.90 | 0.47 | 26.79 | 0.44 | 21.85 | 0.41 |
| **Social group** | | | | | | | | |
| SC | | 2,664 | 35.13 | 0.47 | 25.57 | 0.43 | 21.45 | 0.41 |
| ST | | 1,593 | 29.73 | 0.45 | 23.43 | 0.42 | 19.24 | 0.39 |
| OBC | | 7,314 | 37.81 | 0.48 | 28.69 | 0.45 | 23.31 | 0.42 |
| Others | | 7,330 | 36.29 | 0.48 | 27.61 | 0.44 | 22.02 | 0.41 |
| **Religion** | | | | | | | | |
| Hinduism | | 14,505 | 37.39 | 0.48 | 28.37 | 0.45 | 23.23 | 0.42 |
| Islam | | 2,246 | 30.83 | 0.46 | 23.10 | 0.42 | 18.36 | 0.38 |
| Christian | | 1,265 | 36.38 | 0.48 | 28.43 | 0.45 | 21.56 | 0.41 |
| Others | | 885 | 36.01 | 0.48 | 24.34 | 0.43 | 18.16 | 0.38 |
| **Insurance coverage** | | | | | | | | |
| Government | | 3,486 | 29.31 | 0.45 | 22.01 | 0.41 | 18.46 | 0.39 |
| Private | | 708 | 20.71 | 0.41 | 16.70 | 0.37 | 10.14 | 0.30 |
| Others | | 118 | 20.41 | 0.40 | 16.64 | 0.37 | 11.88 | 0.32 |
| None | | 14,589 | 39.02 | 0.48 | 29.48 | 0.45 | 23.92 | 0.42 |
| **Rank-weighted headcount (%)** | | 18,901 | 43.34 | 0.57 | 33.99 | 0.55 | 28.39 | 0.53 |
| **Concentration Index headcount** | | **Total obs.** | **Index value** | **s.e.*** | **Index value** | **s.e.*** | **Index value** | **s.e.*** |
|  |  | 18,901 | -0.189 | 0.017* | -0.233 | 0.017* | -0.27 | 0.019* |
| **Source:** Author's computation using NSSO 75th round, 2018. | | | | | | | | |
| *p-value<0.01, s.e.- standard error, S.D.- standard deviation | | | | | | | | |

| **Table 14: Inpatient intensity of catastrophic health expenditure of elderly population (Tendulkar)** | | | | | | | | |
| --- | --- | --- | --- | --- | --- | --- | --- | --- |
| **CHE as a share of household usual consumption expenditure (%)** | | | | | | | | |
| **Threshold (%)** | | | **20%** | | **30%** | | **40%** | |
| **Overshoot (%)** | | | | | | | | |
|  |  | **Total obs.** | **Mean (%)** | **S.D.** | **Mean (%)** | **S.D.** | **Mean (%)** | **S.D.** |
|  |  | 18,901 | 41.06 | 4.50 | 37.89 | 4.49 | 35.41 | 4.49 |
| **Economic quantile** | | | | | | | | |
| Poorest | | 2,832 | 131.51 | 10.29 | 126.84 | 10.29 | 122.87 | 10.28 |
| Poor | | 3,030 | 32.70 | 1.13 | 29.12 | 1.11 | 26.16 | 1.09 |
| Middle | | 3,338 | 22.04 | 0.72 | 18.93 | 0.70 | 16.51 | 0.68 |
| Rich | | 4,306 | 16.87 | 0.66 | 14.29 | 0.64 | 12.44 | 0.63 |
| Richest | | 5,395 | 13.71 | 0.61 | 11.42 | 0.59 | 9.84 | 0.58 |
| **Sector** | | | | | | | | |
| Rural | | 10,146 | 51.19 | 5.56 | 47.82 | 5.55 | 45.13 | 5.54 |
| Urban | | 8,755 | 23.02 | 1.15 | 20.20 | 1.14 | 18.10 | 1.13 |
| **Sex** | | | | | | | | |
| Male | | 14,765 | 42.42 | 5.04 | 39.25 | 5.04 | 36.76 | 5.03 |
| Female | | 4,134 | 36.36 | 1.54 | 33.19 | 1.53 | 30.75 | 1.51 |
| **Social group** | | | | | | | | |
| SC | | 2,664 | 37.12 | 2.05 | 34.15 | 2.04 | 31.79 | 2.03 |
| ST | | 1,593 | 40.11 | 1.91 | 37.47 | 1.89 | 35.39 | 1.88 |
| OBC | | 7,314 | 51.94 | 6.62 | 48.61 | 6.61 | 46.04 | 6.61 |
| Others | | 7,330 | 30.15 | 1.34 | 27.01 | 1.32 | 24.53 | 1.31 |
| **Religion** | | | | | | | | |
| Hinduism | | 14,505 | 45.80 | 5.03 | 42.53 | 5.02 | 39.98 | 5.02 |
| Islam | | 2,246 | 23.12 | 0.90 | 20.47 | 0.89 | 18.39 | 0.87 |
| Christian | | 1,265 | 20.74 | 0.64 | 17.57 | 0.62 | 15.01 | 0.60 |
| Others | | 885 | 26.77 | 2.09 | 23.81 | 2.08 | 21.75 | 2.07 |
| **Insurance coverage** | | | | | | | | |
| Government | | 3,486 | 27.97 | 1.37 | 25.45 | 1.35 | 23.43 | 1.34 |
| Private | | 708 | 11.08 | 0.47 | 9.24 | 0.45 | 7.87 | 0.43 |
| Others | | 118 | 16.13 | 0.73 | 14.36 | 0.72 | 12.81 | 0.70 |
| None | | 14,589 | 45.80 | 5.08 | 42.40 | 5.08 | 39.75 | 5.07 |
| **Rank-weighted overshoot (%)** | | 18,901 | 60.27 | 6.61 | 56.64 | 6.72 | 53.70 | 6.81 |
| **Mean positive overshoot (%)** | | 18,901 | 41.06 | 4.50 | 37.89 | 4.49 | 35.41 | 4.49 |
| **Concentration Index overshoot** | | **Total obs.** | **Index value** | **s.e.*** | **Index value** | **s.e.*** | **Index value** | **s.e.*** |
|  |  | 18,901 | -0.468 | 0.091* | -0.495 | 0.099* | -0.516 | 0.106* |
| **Source:** Author's computation using NSSO 75th round, 2018. | | | | | | | | |
| *p-value<0.01, s.e.- standard error, S.D.- standard deviation | | | | | | | | |

**Tables 15 and 16 report the incidence and intensity of CHE at non-subsistence expenditure (Rangarajan committee poverty line) due to inpatient services respectively.**

| **Table 15: Inpatient incidence of catastrophic health expenditure of elderly population (Rangarajan)** | | | | | | | | |
| --- | --- | --- | --- | --- | --- | --- | --- | --- |
| **CHE as a share of household usual consumption expenditure (%)** | | | | | | | | |
| **Threshold (%)** | | | **20%** | | **30%** | | **40%** | |
| **Headcount (%)** | | | | | | | | |
|  |  | **Total obs.** | **Mean (%)** | **S.D.** | **Mean (%)** | **S.D.** | **Mean (%)** | **S.D.** |
|  |  | 18,901 | 37.92 | 0.48 | 29.92 | 0.45 | 24.49 | 0.43 |
| **Economic quantile** | | | | | | | | |
| Poorest | | 2,832 | 38.41 | 0.48 | 34.59 | 0.47 | 30.17 | 0.45 |
| Poor | | 3,030 | 46.64 | 0.50 | 39.05 | 0.48 | 34.28 | 0.47 |
| Middle | | 3,338 | 40.94 | 0.49 | 33.15 | 0.47 | 27.52 | 0.44 |
| Rich | | 4,306 | 35.99 | 0.48 | 25.31 | 0.43 | 19.33 | 0.39 |
| Richest | | 5,395 | 30.55 | 0.46 | 21.18 | 0.40 | 15.15 | 0.35 |
| **Sector** | | | | | | | | |
| Rural | | 10,146 | 39.06 | 0.48 | 31.23 | 0.46 | 25.94 | 0.43 |
| Urban | | 8,755 | 35.91 | 0.47 | 27.59 | 0.44 | 21.92 | 0.41 |
| **Sex** | | | | | | | | |
| Male | | 14,765 | 38.01 | 0.48 | 29.82 | 0.45 | 24.57 | 0.43 |
| Female | | 4,134 | 37.64 | 0.48 | 30.27 | 0.45 | 24.23 | 0.42 |
| **Social group** | | | | | | | | |
| SC | | 2,664 | 34.23 | 0.47 | 26.78 | 0.44 | 22.33 | 0.41 |
| ST | | 1,593 | 32.43 | 0.46 | 26.08 | 0.43 | 20.78 | 0.40 |
| OBC | | 7,314 | 38.93 | 0.48 | 30.74 | 0.46 | 24.97 | 0.43 |
| Others | | 7,330 | 39.03 | 0.48 | 30.80 | 0.46 | 25.34 | 0.43 |
| **Religion** | | | | | | | | |
| Hinduism | | 14,505 | 38.65 | 0.48 | 30.61 | 0.46 | 25.14 | 0.43 |
| Islam | | 2,246 | 33.12 | 0.47 | 26.06 | 0.43 | 21.13 | 0.40 |
| Christian | | 1,265 | 40.49 | 0.49 | 31.09 | 0.46 | 25.54 | 0.43 |
| Others | | 885 | 35.90 | 0.47 | 27.05 | 0.44 | 20.81 | 0.40 |
| **Insurance coverage** | | | | | | | | |
| Government | | 3,486 | 32.65 | 0.46 | 24.52 | 0.43 | 19.95 | 0.39 |
| Private | | 708 | 22.07 | 0.41 | 18.75 | 0.39 | 15.07 | 0.35 |
| Others | | 118 | 25.13 | 0.43 | 18.49 | 0.38 | 15.93 | 0.36 |
| None | | 14,589 | 40.02 | 0.48 | 31.84 | 0.46 | 26.09 | 0.43 |
| **Rank-weighted headcount (%)** | | 18,901 | 41.63 | 0.53 | 34.75 | 0.53 | 29.44 | 0.51 |
| **Concentration Index headcount** | | **Total obs.** | **Index value** | **s.e.*** | **Index value** | **s.e.*** | **Index value** | **s.e.*** |
|  |  | 18,901 | -0.097 | 0.017* | -0.161 | 0.018* | -0.201 | 0.018* |
| **Source:** Author's computation using NSSO 75th round, 2018. | | | | | | | | |
| *p-value<0.01, s.e.- standard error, S.D.- standard deviation | | | | | | | | |

| **Table 16: Inpatient intensity of catastrophic health expenditure of elderly population (Rangarajan)** | | | | | | | | |
| --- | --- | --- | --- | --- | --- | --- | --- | --- |
| **CHE as a share of household usual consumption expenditure (%)** | | | | | | | | |
| **Threshold (%)** | | | **20%** | | **30%** | | **40%** | |
| **Overshoot (%)** | | | | | | | | |
|  |  | **Total obs.** | **Mean (%)** | **S.D.** | **Mean (%)** | **S.D.** | **Mean (%)** | **S.D.** |
|  |  | 18,901 | 57.17 | 3.92 | 53.81 | 3.91 | 51.10 | 3.90 |
| **Economic quantile** | | | | | | | | |
| Poorest | | 2,832 | 159.17 | 7.92 | 155.53 | 7.91 | 152.27 | 7.90 |
| Poor | | 3,030 | 74.43 | 4.13 | 70.16 | 4.12 | 66.50 | 4.11 |
| Middle | | 3,338 | 33.13 | 1.10 | 29.44 | 1.08 | 26.40 | 1.06 |
| Rich | | 4,306 | 22.16 | 0.83 | 19.12 | 0.81 | 16.90 | 0.79 |
| Richest | | 5,395 | 15.70 | 0.67 | 13.15 | 0.65 | 11.40 | 0.64 |
| **Sector** | | | | | | | | |
| Rural | | 10,146 | 66.48 | 4.37 | 62.99 | 4.36 | 60.13 | 4.35 |
| Urban | | 8,755 | 40.60 | 2.93 | 37.46 | 2.92 | 35.02 | 2.91 |
| **Sex** | | | | | | | | |
| Male | | 14,765 | 58.76 | 4.12 | 55.39 | 4.11 | 52.68 | 4.11 |
| Female | | 4,134 | 51.72 | 3.09 | 48.35 | 3.08 | 45.64 | 3.07 |
| **Social group** | | | | | | | | |
| SC | | 2,664 | 48.87 | 2.38 | 45.87 | 2.36 | 43.40 | 2.35 |
| ST | | 1,593 | 44.30 | 3.95 | 41.38 | 3.94 | 39.09 | 3.93 |
| OBC | | 7,314 | 67.28 | 4.92 | 63.82 | 4.91 | 61.04 | 4.90 |
| Others | | 7,330 | 50.59 | 3.01 | 47.11 | 3.01 | 44.32 | 2.99 |
| **Religion** | | | | | | | | |
| Hinduism | | 14,505 | 61.33 | 4.15 | 57.89 | 4.14 | 55.12 | 4.13 |
| Islam | | 2,246 | 48.44 | 3.33 | 45.52 | 3.32 | 43.13 | 3.31 |
| Christian | | 1,265 | 35.77 | 2.36 | 32.32 | 2.35 | 29.53 | 2.34 |
| Others | | 885 | 21.90 | 0.96 | 18.72 | 0.95 | 16.29 | 0.93 |
| **Insurance coverage** | | | | | | | | |
| Government | | 3,486 | 44.55 | 3.40 | 41.74 | 3.39 | 39.55 | 3.38 |
| Private | | 708 | 18.70 | 0.80 | 16.66 | 0.78 | 15.01 | 0.76 |
| Others | | 118 | 15.80 | 0.62 | 13.83 | 0.60 | 12.13 | 0.58 |
| None | | 14,589 | 62.27 | 4.12 | 58.70 | 4.12 | 55.81 | 4.11 |
| **Rank-weighted overshoot (%)** | | 18,901 | 83.38 | 5.71 | 79.72 | 5.79 | 76.64 | 5.85 |
| **Mean positive overshoot (%)** | | 18,901 | 57.17 | 3.92 | 53.81 | 3.91 | 51.10 | 3.90 |
| **Concentration Index overshoot** | | **Total obs.** | **Index value** | **s.e.*** | **Index value** | **s.e.*** | **Index value** | **s.e.*** |
|  |  | 18,901 | -0.458 | 0.059* | -0.481 | 0.063* | -0.499 | 0.066* |
| **Source:** Author's computation using NSSO 75th round, 2018. | | | | | | | | |
| *p-value<0.01, s.e.- standard error, S.D.- standard deviation | | | | | | | | |

**Tables 17 and 18 report the incidence and intensity of CHE at subsistence expenditure due to outpatient services respectively.**

| **Table 17: Outpatient incidence of catastrophic health expenditure of elderly population** | | | | | | | | | | |
| --- | --- | --- | --- | --- | --- | --- | --- | --- | --- | --- |
| **CHE as a share of household usual consumption expenditure (%)** | | | | | | | | | | |
| **Threshold (%)** | | | **10%** | | **20%** | | **30%** | | **40%** | |
| **Headcount (%)** | | | | | | | | | | |
|  |  | **Total obs.** | **Mean (%)** | **S.D.** | **Mean (%)** | **S.D.** | **Mean (%)** | **S.D.** | **Mean (%)** | **S.D.** |
|  |  | 11,084 | 43.56 | 0.49 | 24.74 | 0.43 | 16.16 | 0.36 | 11.84 | 0.32 |
| **Economic quantile** | | | | | | | | | | |
| Poorest | | 1,200 | 58.11 | 0.49 | 38.40 | 0.48 | 29.82 | 0.45 | 23.78 | 0.42 |
| Poor | | 1,499 | 52.12 | 0.49 | 33.02 | 0.47 | 18.86 | 0.39 | 13.43 | 0.34 |
| Middle | | 1,766 | 45.25 | 0.49 | 24.23 | 0.42 | 15.42 | 0.36 | 10.06 | 0.30 |
| Rich | | 2,567 | 40.00 | 0.48 | 21.56 | 0.41 | 14.44 | 0.35 | 10.51 | 0.31 |
| Richest | | 4,052 | 33.39 | 0.47 | 16.00 | 0.36 | 9.58 | 0.29 | 6.98 | 0.25 |
| **Sector** | | | | | | | | | | |
| Rural | | 5,457 | 48.58 | 0.49 | 28.96 | 0.45 | 19.13 | 0.39 | 14.18 | 0.34 |
| Urban | | 5,627 | 35.97 | 0.47 | 18.35 | 0.38 | 11.66 | 0.32 | 8.31 | 0.27 |
| **Sex** | | | | | | | | | | |
| Male | | 7,527 | 45.10 | 0.49 | 25.57 | 0.43 | 16.34 | 0.36 | 11.70 | 0.32 |
| Female | | 3,555 | 40.39 | 0.49 | 23.03 | 0.42 | 15.78 | 0.36 | 12.13 | 0.32 |
| **Social group** | | | | | | | | | | |
| SC | | 1,460 | 51.67 | 0.49 | 33.06 | 0.47 | 22.37 | 0.41 | 15.45 | 0.36 |
| ST | | 550 | 52.87 | 0.49 | 34.98 | 0.47 | 24.53 | 0.43 | 15.52 | 0.36 |
| OBC | | 4,133 | 42.31 | 0.49 | 23.40 | 0.42 | 15.06 | 0.35 | 11.01 | 0.31 |
| Others | | 4,941 | 40.42 | 0.49 | 21.51 | 0.41 | 13.76 | 0.34 | 10.77 | 0.31 |
| **Religion** | | | | | | | | | | |
| Hinduism | | 8,278 | 44.87 | 0.49 | 25.72 | 0.43 | 17.03 | 0.37 | 12.68 | 0.33 |
| Islam | | 1,538 | 42.99 | 0.49 | 21.48 | 0.41 | 12.14 | 0.32 | 7.69 | 0.26 |
| Christian | | 725 | 29.74 | 0.45 | 15.44 | 0.36 | 9.15 | 0.28 | 5.64 | 0.23 |
| Others | | 543 | 34.80 | 0.47 | 25.55 | 0.43 | 18.72 | 0.39 | 14.57 | 0.35 |
| **Insurance coverage** | | | | | | | | | | |
| Government | | 2,431 | 38.13 | 0.48 | 20.63 | 0.40 | 13.80 | 0.34 | 8.23 | 0.27 |
| Private | | 571 | 28.84 | 0.45 | 11.08 | 0.31 | 9.01 | 0.28 | 7.47 | 0.26 |
| Others | | 79 | 32.64 | 0.46 | 21.57 | 0.41 | 16.21 | 0.36 | 9.87 | 0.29 |
| None | | 8,003 | 45.97 | 0.49 | 26.68 | 0.44 | 17.23 | 0.37 | 13.14 | 0.33 |
| **Rank-weighted headcount (%)** | | 11,084 | 52.37 | 0.59 | 30.64 | 0.53 | 20.24 | 0.46 | 14.89 | 0.40 |
| **Concentration Index headcount** | | **Total obs.** | **Index value** | **s.e.*** | **Index value** | **s.e.*** | **Index value** | **s.e.*** | **Index value** | **s.e.*** |
|  | | 11,084 | -0.202 | 0.026* | -0.238 | 0.031* | -0.252 | 0.037* | -0.257 | 0.043* |
| **Source:** Author's computation using NSSO 75th round, 2018. | | | | | | | | | | |
| *p-value<0.01, s.e.- standard error, S.D.- standard deviation | | | | | | | | | | |

| **Table 18: Outpatient intensity of catastrophic health expenditure of elderly population** | | | | | | | | | | |
| --- | --- | --- | --- | --- | --- | --- | --- | --- | --- | --- |
| **CHE as a share of household usual consumption expenditure (%)** | | | | | | | | | | |
| **Threshold (%)** | | | **10%** | | **20%** | | **30%** | | **40%** | |
| **Overshoot (%)** | | | | | | | | | | |
|  |  | **Total obs.** | **Mean (%)** | **S.D.** | **Mean (%)** | **S.D.** | **Mean (%)** | **S.D.** | **Mean (%)** | **S.D.** |
|  |  | 11,084 | 13.58 | 0.51 | 10.36 | 0.50 | 8.38 | 0.48 | 7.02 | 0.47 |
| **Economic quantile** | | | | | | | | | | |
| Poorest | | 1,200 | 27.01 | 0.74 | 22.50 | 0.72 | 19.20 | 0.70 | 16.51 | 0.68 |
| Poor | | 1,499 | 18.56 | 0.65 | 14.42 | 0.64 | 11.89 | 0.62 | 10.37 | 0.61 |
| Middle | | 1,766 | 12.02 | 0.50 | 8.89 | 0.49 | 6.92 | 0.47 | 5.72 | 0.46 |
| Rich | | 2,567 | 11.02 | 0.46 | 8.06 | 0.44 | 6.34 | 0.43 | 5.12 | 0.42 |
| Richest | | 4,052 | 6.88 | 0.26 | 4.57 | 0.24 | 3.34 | 0.23 | 2.54 | 0.21 |
| **Sector** | | | | | | | | | | |
| Rural | | 5,457 | 16.18 | 0.58 | 12.52 | 0.56 | 10.18 | 0.54 | 8.57 | 0.53 |
| Urban | | 5,627 | 9.66 | 0.40 | 7.09 | 0.38 | 5.65 | 0.37 | 4.68 | 0.36 |
| **Sex** | | | | | | | | | | |
| Male | | 7,527 | 12.59 | 0.46 | 9.29 | 0.44 | 7.29 | 0.43 | 5.91 | 0.42 |
| Female | | 3,555 | 15.61 | 0.61 | 12.55 | 0.59 | 10.62 | 0.58 | 9.30 | 0.56 |
| **Social group** | | | | | | | | | | |
| SC | | 1,460 | 23.04 | 0.91 | 18.97 | 0.90 | 16.36 | 0.89 | 14.56 | 0.87 |
| ST | | 550 | 16.88 | 0.39 | 12.76 | 0.37 | 9.83 | 0.35 | 7.94 | 0.33 |
| OBC | | 4,133 | 13.09 | 0.45 | 9.98 | 0.43 | 8.07 | 0.41 | 6.80 | 0.40 |
| Others | | 4,941 | 9.73 | 0.31 | 6.86 | 0.29 | 5.17 | 0.27 | 3.97 | 0.25 |
| **Religion** | | | | | | | | | | |
| Hinduism | | 8,278 | 13.81 | 0.48 | 10.46 | 0.47 | 8.40 | 0.45 | 6.96 | 0.44 |
| Islam | | 1,538 | 10.14 | 0.36 | 7.26 | 0.34 | 5.51 | 0.33 | 4.58 | 0.31 |
| Christian | | 725 | 9.55 | 0.46 | 7.48 | 0.45 | 6.32 | 0.43 | 5.69 | 0.42 |
| Others | | 543 | 24.36 | 1.17 | 21.40 | 1.16 | 19.30 | 1.14 | 17.64 | 1.13 |
| **Insurance coverage** | | | | | | | | | | |
| Government | | 2,431 | 12.04 | 0.55 | 9.29 | 0.54 | 7.66 | 0.52 | 6.62 | 0.51 |
| Private | | 571 | 6.89 | 0.20 | 5.04 | 0.18 | 4.08 | 0.15 | 3.21 | 0.13 |
| Others | | 79 | 7.28 | 0.15 | 4.75 | 0.11 | 2.87 | 0.08 | 1.50 | 0.06 |
| None | | 8,003 | 14.41 | 0.51 | 10.98 | 0.50 | 8.84 | 0.48 | 7.36 | 0.47 |
| **Rank-weighted overshoot (%)** | | 11,084 | 17.21 | 0.65 | 13.54 | 0.65 | 11.17 | 0.65 | 9.51 | 0.64 |
| **Mean positive overshoot (%)** | | 11,084 | 13.58 | 0.51 | 10.36 | 0.50 | 8.38 | 0.48 | 7.02 | 0.47 |
| **Concentration Index overshoot** | | **Total obs.** | **Index value** | **s.e.*** | **Index value** | **s.e.*** | **Index value** | **s.e.*** | **Index value** | **s.e.*** |
|  | | 11,084 | -0.267 | 0.056* | -0.306 | 0.071* | -0.333 | 0.085* | -0.354 | 0.099* |
| **Source:** Author's computation using NSSO 75th round, 2018. | | | | | | | | | | |
| *p-value<0.01, s.e.- standard error, S.D.- standard deviation | | | | | | | | | | |

**Tables 19 and 20 report the incidence and intensity of CHE at non-subsistence expenditure (Tendulkar committee poverty line) due to outpatient services respectively.**

| **Table 19: Outpatient incidence of catastrophic health expenditure of elderly population (Tendulkar)** | | | | | | | | |
| --- | --- | --- | --- | --- | --- | --- | --- | --- |
| **CHE as a share of household usual consumption expenditure (%)** | | | | | | | | |
| **Threshold (%)** | | | **20%** | | **30%** | | **40%** | |
| **Headcount (%)** | | | | | | | | |
|  |  | **Total obs.** | **Mean (%)** | **S.D.** | **Mean (%)** | **S.D.** | **Mean (%)** | **S.D.** |
|  |  | 11,084 | 38.91 | 0.48 | 29.05 | 0.45 | 23.28 | 0.42 |
| **Economic quantile** | | | | | | | | |
| Poorest | | 1,200 | 59.77 | 0.49 | 56.06 | 0.49 | 48.69 | 0.49 |
| Poor | | 1,499 | 57.62 | 0.49 | 44.10 | 0.49 | 36.28 | 0.48 |
| Middle | | 1,766 | 41.12 | 0.49 | 27.90 | 0.44 | 23.01 | 0.42 |
| Rich | | 2,567 | 33.05 | 0.47 | 22.06 | 0.41 | 16.27 | 0.36 |
| Richest | | 4,052 | 21.39 | 0.41 | 13.08 | 0.33 | 8.80 | 0.28 |
| **Sector** | | | | | | | | |
| Rural | | 5,457 | 45.52 | 0.49 | 35.33 | 0.47 | 29.38 | 0.45 |
| Urban | | 5,627 | 28.92 | 0.45 | 19.54 | 0.39 | 14.06 | 0.34 |
| **Sex** | | | | | | | | |
| Male | | 7,527 | 40.68 | 0.49 | 30.38 | 0.45 | 24.24 | 0.42 |
| Female | | 3,555 | 35.27 | 0.47 | 26.32 | 0.44 | 21.31 | 0.40 |
| **Social group** | | | | | | | | |
| SC | | 1,460 | 47.07 | 0.49 | 38.70 | 0.48 | 31.93 | 0.46 |
| ST | | 550 | 50.16 | 0.49 | 43.92 | 0.49 | 35.17 | 0.47 |
| OBC | | 4,133 | 40.58 | 0.49 | 29.38 | 0.45 | 23.07 | 0.42 |
| Others | | 4,941 | 32.71 | 0.46 | 23.16 | 0.42 | 18.65 | 0.38 |
| **Religion** | | | | | | | | |
| Hinduism | | 8,278 | 40.14 | 0.49 | 29.80 | 0.45 | 23.74 | 0.42 |
| Islam | | 1,538 | 39.06 | 0.48 | 30.17 | 0.45 | 25.03 | 0.43 |
| Christian | | 725 | 24.37 | 0.42 | 15.63 | 0.36 | 12.20 | 0.32 |
| Others | | 543 | 30.57 | 0.46 | 26.69 | 0.44 | 22.36 | 0.41 |
| **Insurance coverage** | | | | | | | | |
| Government | | 2,431 | 34.35 | 0.47 | 23.56 | 0.42 | 19.99 | 0.39 |
| Private | | 571 | 17.87 | 0.38 | 13.86 | 0.34 | 9.16 | 0.28 |
| Others | | 79 | 19.01 | 0.39 | 18.40 | 0.38 | 12.94 | 0.33 |
| None | | 8,003 | 41.43 | 0.49 | 31.50 | 0.46 | 25.03 | 0.43 |
| **Rank-weighted headcount (%)** | | 11,084 | 52.58 | 0.65 | 40.90 | 0.63 | 33.36 | 0.60 |
| **Concentration Index headcount** | | **Total obs.** | **Index value** | **s.e.*** | **Index value** | **s.e.*** | **Index value** | **s.e.*** |
|  |  | 11,084 | -0.351 | 0.025* | -0.407 | 0.028* | -0.432 | 0.031* |
| **Source:** Author's computation using NSSO 75th round, 2018. | | | | | | | | |
| *p-value<0.01, s.e.- standard error, S.D.- standard deviation | | | | | | | | |

| **Table 20: Outpatient intensity of catastrophic health expenditure of elderly population (Tendulkar)** | | | | | | | | |
| --- | --- | --- | --- | --- | --- | --- | --- | --- |
| **CHE as a share of household usual consumption expenditure (%)** | | | | | | | | |
| **Threshold (%)** | | | **20%** | | **30%** | | **40%** | |
| **Overshoot (%)** | | | | | | | | |
|  |  | **Total obs.** | **Mean (%)** | **S.D.** | **Mean (%)** | **S.D.** | **Mean (%)** | **S.D.** |
|  |  | 11,084 | 52.46 | 3.45 | 49.12 | 3.44 | 46.52 | 3.43 |
| **Economic quantile** | | | | | | | | |
| Poorest | | 1,200 | 241.06 | 8.44 | 235.25 | 8.42 | 230.06 | 8.41 |
| Poor | | 1,499 | 44.56 | 1.41 | 39.46 | 1.39 | 35.37 | 1.38 |
| Middle | | 1,766 | 21.77 | 0.98 | 18.48 | 0.97 | 15.98 | 0.95 |
| Rich | | 2,567 | 15.32 | 0.68 | 12.61 | 0.66 | 10.75 | 0.65 |
| Richest | | 4,052 | 6.82 | 0.31 | 5.17 | 0.29 | 4.09 | 0.27 |
| **Sector** | | | | | | | | |
| Rural | | 5,457 | 74.94 | 4.37 | 70.94 | 4.36 | 67.70 | 4.35 |
| Urban | | 5,627 | 18.48 | 0.88 | 16.12 | 0.86 | 14.48 | 0.85 |
| **Sex** | | | | | | | | |
| Male | | 7,527 | 54.69 | 3.80 | 51.19 | 3.79 | 48.47 | 3.78 |
| Female | | 3,555 | 47.91 | 2.60 | 44.87 | 2.58 | 42.52 | 2.57 |
| **Social group** | | | | | | | | |
| SC | | 1,460 | 121.65 | 5.76 | 117.3 | 5.75 | 113.79 | 5.74 |
| ST | | 550 | 58.21 | 4.66 | 53.52 | 4.66 | 49.37 | 4.65 |
| OBC | | 4,133 | 53.13 | 3.56 | 49.70 | 3.55 | 47.09 | 3.54 |
| Others | | 4,941 | 22.07 | 0.94 | 19.36 | 0.92 | 17.31 | 0.91 |
| **Religion** | | | | | | | | |
| Hinduism | | 8,278 | 53.15 | 3.45 | 49.70 | 3.44 | 47.04 | 3.43 |
| Islam | | 1,538 | 48.24 | 2.68 | 44.86 | 2.67 | 42.17 | 2.66 |
| Christian | | 725 | 15.62 | 0.64 | 13.66 | 0.62 | 12.27 | 0.60 |
| Others | | 543 | 98.14 | 6.38 | 95.37 | 6.37 | 92.99 | 6.36 |
| **Insurance coverage** | | | | | | | | |
| Government | | 2,431 | 47.94 | 3.67 | 45.09 | 3.66 | 42.97 | 3.65 |
| Private | | 571 | 13.50 | 2.56 | 12.04 | 2.56 | 10.95 | 2.55 |
| Others | | 79 | 14.51 | 0.42 | 12.66 | 0.39 | 11.21 | 0.37 |
| None | | 8,003 | 56.00 | 3.43 | 52.40 | 3.42 | 49.58 | 3.41 |
| **Rank-weighted overshoot (%)** | | 11,084 | 85.84 | 5.64 | 81.64 | 5.72 | 78.23 | 5.77 |
| **Mean positive overshoot (%)** | | 11,084 | 52.46 | 3.45 | 49.12 | 3.44 | 46.52 | 3.43 |
| **Concentration Index overshoot** | | **Total obs.** | **Index value** | **s.e.*** | **Index value** | **s.e.*** | **Index value** | **s.e.*** |
|  |  | 11,084 | -0.636 | 0.114* | -0.662 | 0.122* | -0.681 | 0.128* |
| **Source:** Author's computation using NSSO 75th round, 2018. | | | | | | | | |
| *p-value<0.01, s.e.- standard error, S.D.- standard deviation | | | | | | | | |

**Tables 21 and 22 report the incidence and intensity of CHE at non-subsistence expenditure (Rangarajan committee poverty line) due to outpatient services respectively.**

| **Table 21: Outpatient incidence of catastrophic health expenditure of elderly population (Rangarajan)** | | | | | | | | |
| --- | --- | --- | --- | --- | --- | --- | --- | --- |
| **CHE as a share of household usual consumption expenditure (%)** | | | | | | | | |
| **Threshold (%)** | | | **20%** | | **30%** | | **40%** | |
| **Headcount (%)** | | | | | | | | |
|  |  | **Total obs.** | **Mean (%)** | **S.D.** | **Mean (%)** | **S.D.** | **Mean (%)** | **S.D.** |
|  |  | 11,084 | 41.06 | 0.49 | 31.61 | 0.46 | 25.98 | 0.43 |
| **Economic quantile** | | | | | | | | |
| Poorest | | 1,200 | 44.78 | 0.49 | 43.50 | 0.49 | 40.98 | 0.49 |
| Poor | | 1,499 | 65.55 | 0.47 | 53.58 | 0.49 | 46.24 | 0.49 |
| Middle | | 1,766 | 50.87 | 0.49 | 35.14 | 0.47 | 28.53 | 0.45 |
| Rich | | 2,567 | 36.24 | 0.48 | 27.32 | 0.44 | 19.74 | 0.39 |
| Richest | | 4,052 | 24.12 | 0.42 | 14.87 | 0.35 | 10.56 | 0.31 |
| **Sector** | | | | | | | | |
| Rural | | 5,457 | 46.54 | 0.49 | 36.69 | 0.48 | 31.59 | 0.46 |
| Urban | | 5,627 | 32.78 | 0.46 | 23.92 | 0.42 | 17.49 | 0.38 |
| **Sex** | | | | | | | | |
| Male | | 7,527 | 42.87 | 0.49 | 32.94 | 0.47 | 27.15 | 0.44 |
| Female | | 3,555 | 37.34 | 0.48 | 28.85 | 0.45 | 23.56 | 0.42 |
| **Social group** | | | | | | | | |
| SC | | 1,460 | 46.57 | 0.49 | 39.82 | 0.48 | 35.00 | 0.47 |
| ST | | 550 | 45.44 | 0.49 | 38.72 | 0.48 | 36.93 | 0.48 |
| OBC | | 4,133 | 42.23 | 0.49 | 33.01 | 0.47 | 26.22 | 0.44 |
| Others | | 4,941 | 37.16 | 0.48 | 26.06 | 0.43 | 20.83 | 0.41 |
| **Religion** | | | | | | | | |
| Hinduism | | 8,278 | 42.46 | 0.49 | 32.73 | 0.46 | 26.79 | 0.44 |
| Islam | | 1,538 | 39.71 | 0.48 | 30.47 | 0.46 | 25.92 | 0.43 |
| Christian | | 725 | 28.21 | 0.45 | 18.76 | 0.39 | 14.04 | 0.34 |
| Others | | 543 | 31.51 | 0.46 | 27.43 | 0.44 | 23.94 | 0.42 |
| **Insurance coverage** | | | | | | | | |
| Government | | 2,431 | 36.01 | 0.48 | 26.92 | 0.44 | 20.19 | 0.40 |
| Private | | 571 | 23.17 | 0.42 | 16.02 | 0.36 | 15.22 | 0.35 |
| Others | | 79 | 18.34 | 0.38 | 13.31 | 0.33 | 7.84 | 0.26 |
| None | | 8,003 | 43.57 | 0.49 | 33.87 | 0.47 | 28.31 | 0.45 |
| **Rank-weighted headcount (%)** | | 11,084 | 52.55 | 0.62 | 42.31 | 0.62 | 35.88 | 0.60 |
| **Concentration Index headcount** | | **Total obs.** | **Index value** | **s.e.*** | **Index value** | **s.e.*** | **Index value** | **s.e.*** |
|  |  | 11,084 | -0.279 | 0.027* | -0.338 | 0.028* | -0.380 | 0.031* |
| **Source:** Author's computation using NSSO 75th round, 2018. | | | | | | | | |
| *p-value<0.01, s.e.- standard error, S.D.- standard deviation | | | | | | | | |

| **Table 22: Outpatient intensity of catastrophic health expenditure of elderly population (Rangarajan)** | | | | | | | | |
| --- | --- | --- | --- | --- | --- | --- | --- | --- |
| **CHE as a share of household usual consumption expenditure (%)** | | | | | | | | |
| **Threshold (%)** | | | **20%** | | **30%** | | **40%** | |
| **Overshoot (%)** | | | | | | | | |
|  |  | **Total obs.** | **Mean (%)** | **S.D.** | **Mean (%)** | **S.D.** | **Mean (%)** | **S.D.** |
|  |  | 11,084 | 88.20 | 9.34 | 84.60 | 9.33 | 81.73 | 9.33 |
| **Economic quantile** | | | | | | | | |
| Poorest | | 1,200 | 409.99 | 23.59 | 405.57 | 23.58 | 401.35 | 23.57 |
| Poor | | 1,499 | 86.11 | 3.02 | 80.14 | 3.01 | 75.16 | 2.99 |
| Middle | | 1,766 | 34.42 | 1.61 | 30.10 | 1.60 | 26.96 | 1.59 |
| Rich | | 2,567 | 20.12 | 0.82 | 16.99 | 0.81 | 14.61 | 0.79 |
| Richest | | 4,052 | 8.08 | 0.34 | 6.21 | 0.32 | 4.96 | 0.30 |
| **Sector** | | | | | | | | |
| Rural | | 5,457 | 125.41 | 11.92 | 121.25 | 11.91 | 117.85 | 11.90 |
| Urban | | 5,627 | 31.93 | 1.99 | 29.17 | 1.98 | 27.10 | 1.96 |
| **Sex** | | | | | | | | |
| Male | | 7,527 | 68.02 | 4.73 | 64.27 | 4.72 | 61.30 | 4.75 |
| Female | | 3,555 | 129.55 | 14.83 | 126.26 | 14.83 | 123.59 | 14.82 |
| **Social group** | | | | | | | | |
| SC | | 1,460 | 218.13 | 20.24 | 213.82 | 20.23 | 210.06 | 20.22 |
| ST | | 550 | 153.79 | 8.04 | 149.69 | 8.03 | 145.94 | 8.02 |
| OBC | | 4,133 | 82.23 | 5.55 | 78.48 | 5.54 | 75.51 | 5.53 |
| Others | | 4,941 | 32.61 | 2.74 | 29.49 | 2.73 | 27.18 | 2.72 |
| **Religion** | | | | | | | | |
| Hinduism | | 8,278 | 71.89 | 4.83 | 68.16 | 4.83 | 65.19 | 4.82 |
| Islam | | 1,538 | 72.13 | 4.05 | 68.69 | 4.04 | 65.93 | 4.02 |
| Christian | | 725 | 20.94 | 1.04 | 18.58 | 1.02 | 16.98 | 1.01 |
| Others | | 543 | 577.17 | 42.11 | 574.26 | 42.10 | 571.66 | 42.09 |
| **Insurance coverage** | | | | | | | | |
| Government | | 2,431 | 60.78 | 5.91 | 57.67 | 5.90 | 55.31 | 5.90 |
| Private | | 571 | 10.58 | 0.30 | 8.80 | 0.27 | 7.25 | 0.24 |
| Others | | 79 | 7.38 | 0.24 | 5.96 | 0.21 | 4.89 | 0.19 |
| None | | 8,003 | 100.66 | 10.37 | 96.80 | 10.36 | 93.71 | 10.35 |
| **Rank-weighted overshoot (%)** | | 11,084 | 146.80 | 15.55 | 142.47 | 15.72 | 138.87 | 15.85 |
| **Mean positive overshoot (%)** | | 11,084 | 88.20 | 9.34 | 84.60 | 9.33 | 81.73 | 9.33 |
| **Concentration Index overshoot** | | **Total obs.** | **Index value** | **s.e.*** | **Index value** | **s.e.*** | **Index value** | **s.e.*** |
|  |  | 11,084 | -0.664 | 0.211* | -0.684 | 0.219* | -0.699 | 0.227* |
| **Source:** Author's computation using NSSO 75th round, 2018. | | | | | | | | |
| *p-value<0.01, s.e.- standard error, S.D.- standard deviation | | | | | | | | |
