# Supplementary material for: Incidence and intensity of catastrophic health expenditure and impoverishment among the elderly: an empirical evidence from India
Source: Sci Rep. 2024 Jul 10;14:15908. doi: 10.1038/s41598-024-55142-1 (PMC11237111; doi:10.1038/s41598-024-55142-1)

**Appendix 2:** Supplementary figures

**Figure 9: Concentration curve of incidence of CHE based on subsistence expenditure among older adults (inpatient).**


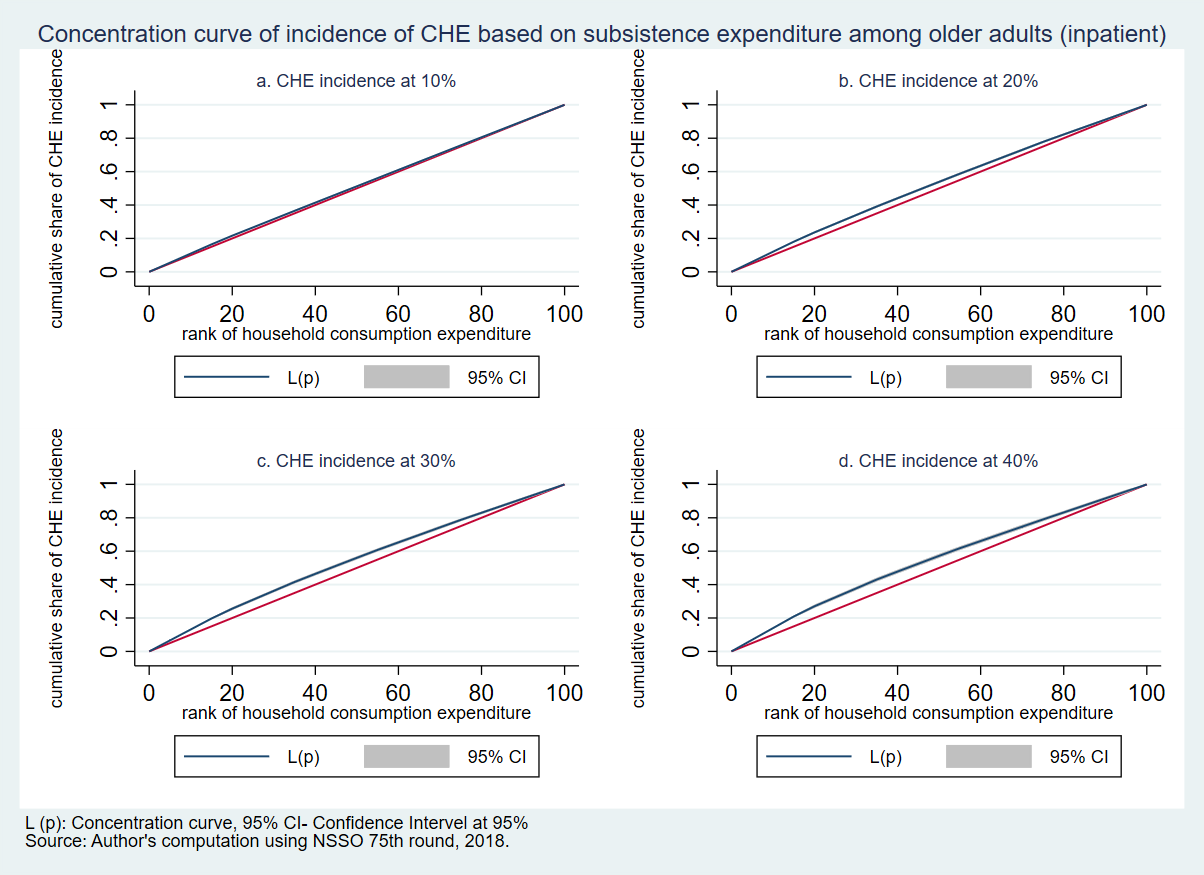


**Figure 10: Concentration curve of intensity of CHE based on subsistence expenditure among older adults (inpatient).**


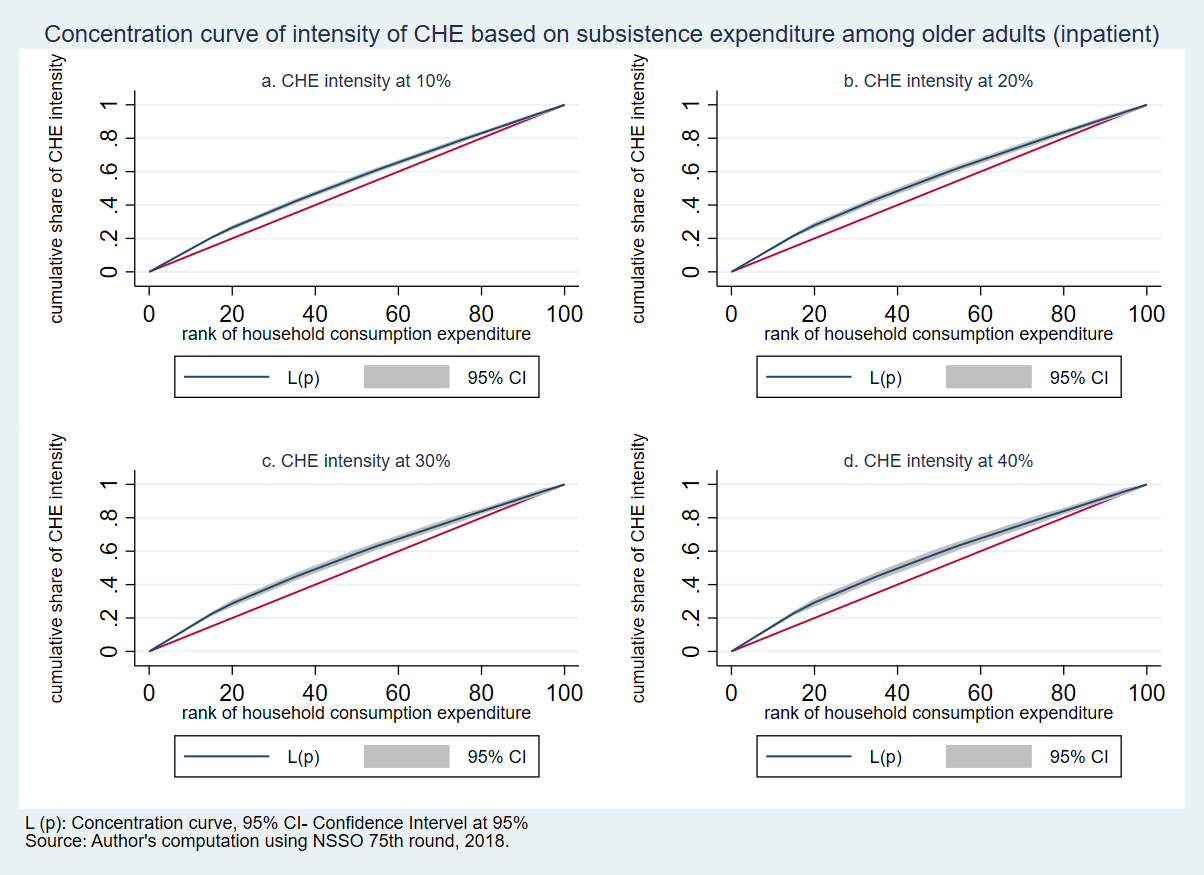


**Figure 11: Concentration curve of incidence of CHE based on non-subsistence expenditure (Tendulkar) among older adults (inpatient).**


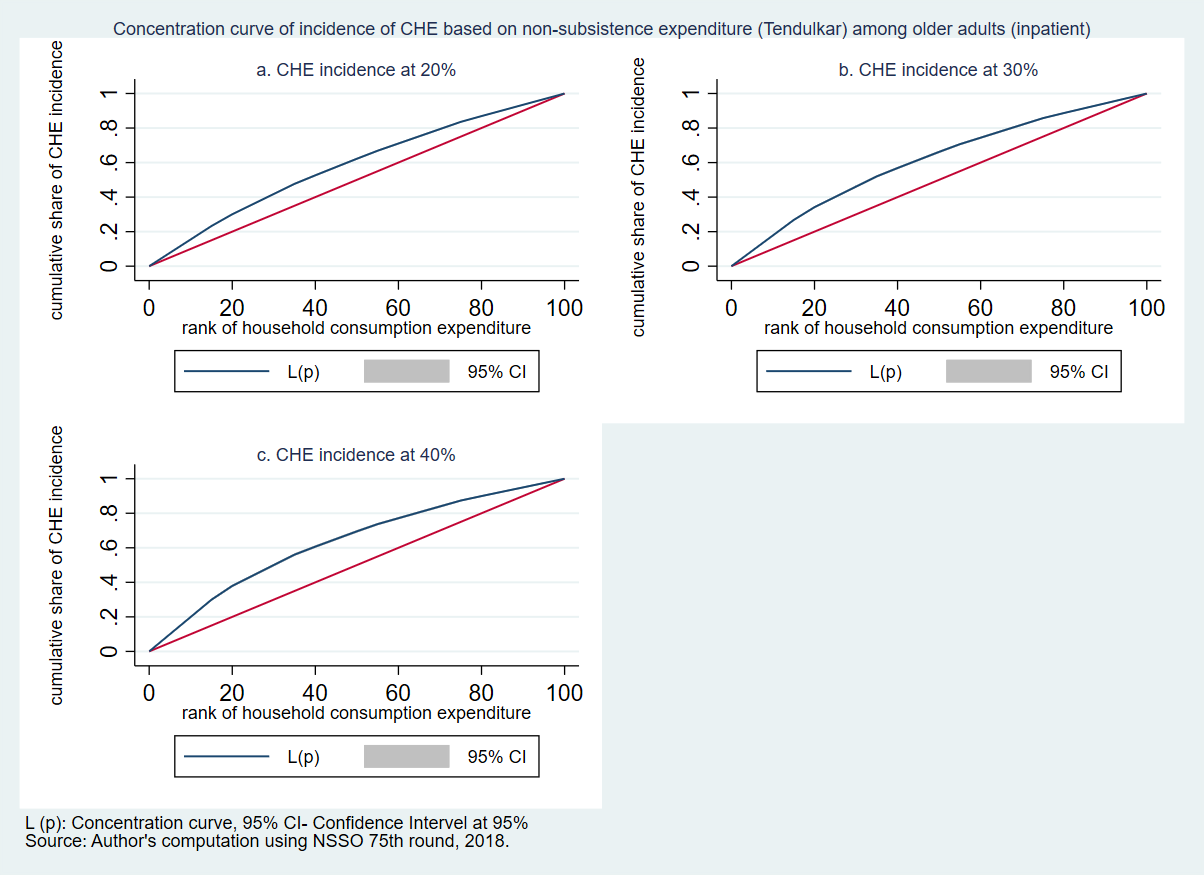


**Figure 12: Concentration curve of intensity of CHE based on non-subsistence expenditure (Tendulkar) among older adults (inpatient).**


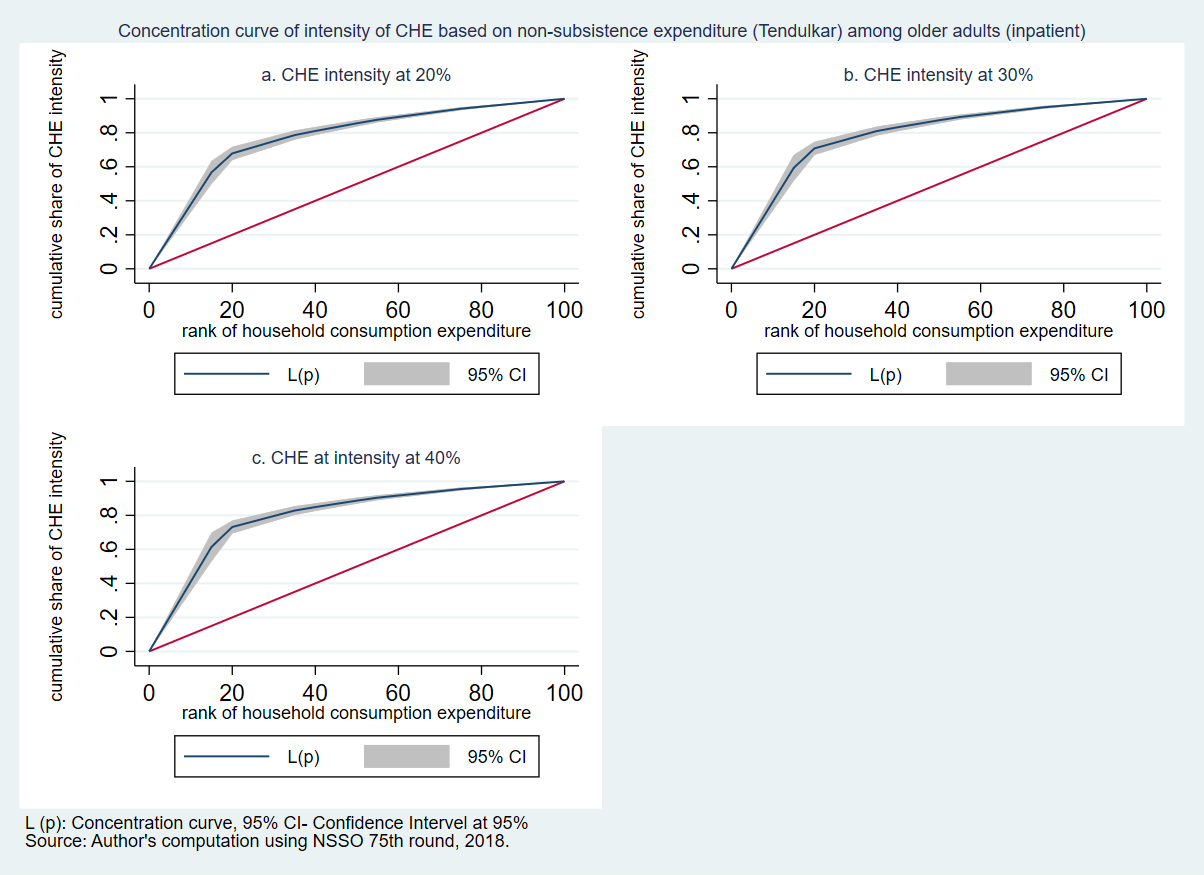


**Figure 13: Concentration curve of incidence of CHE based on non-subsistence expenditure (Rangarajan) among older adults (inpatient).**


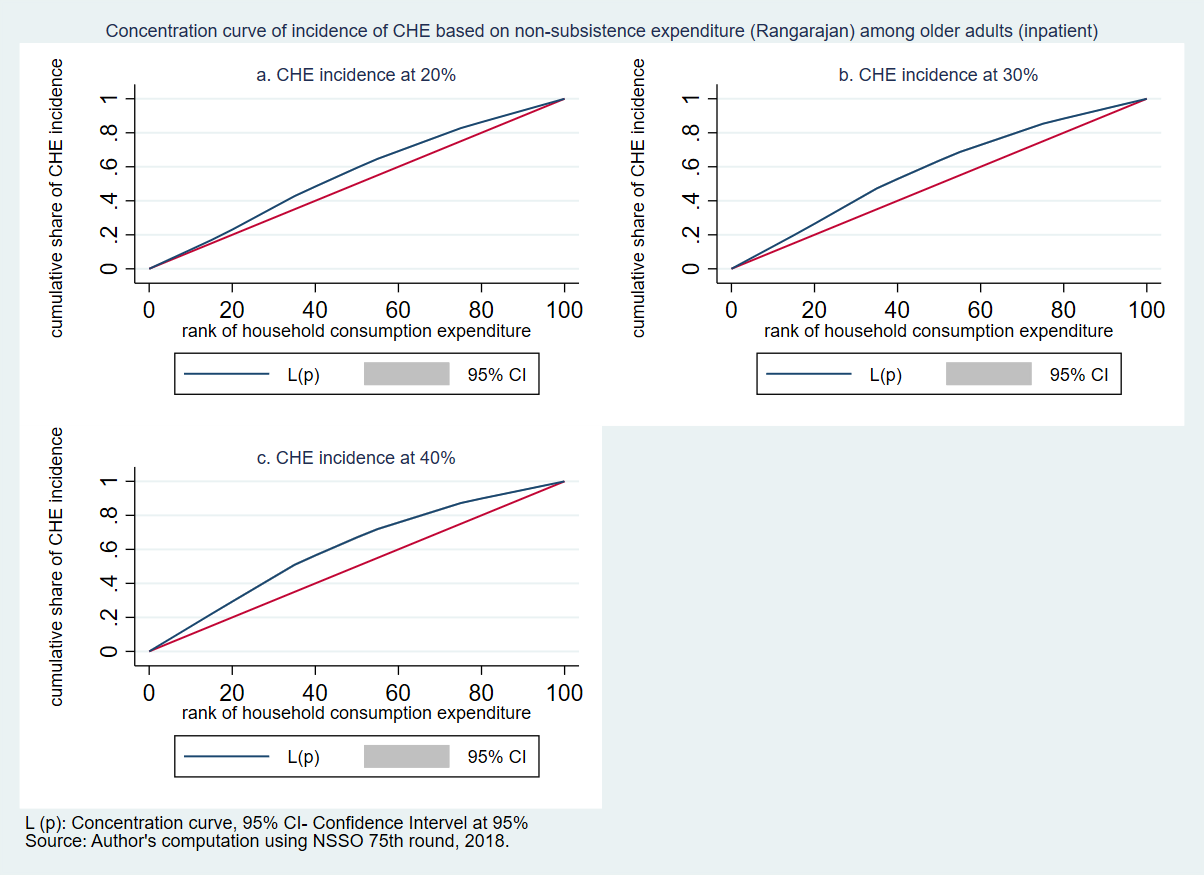


**Figure 14: Concentration curve of intensity of CHE based on non-subsistence expenditure (Rangarajan) among older adults (inpatient).**

**
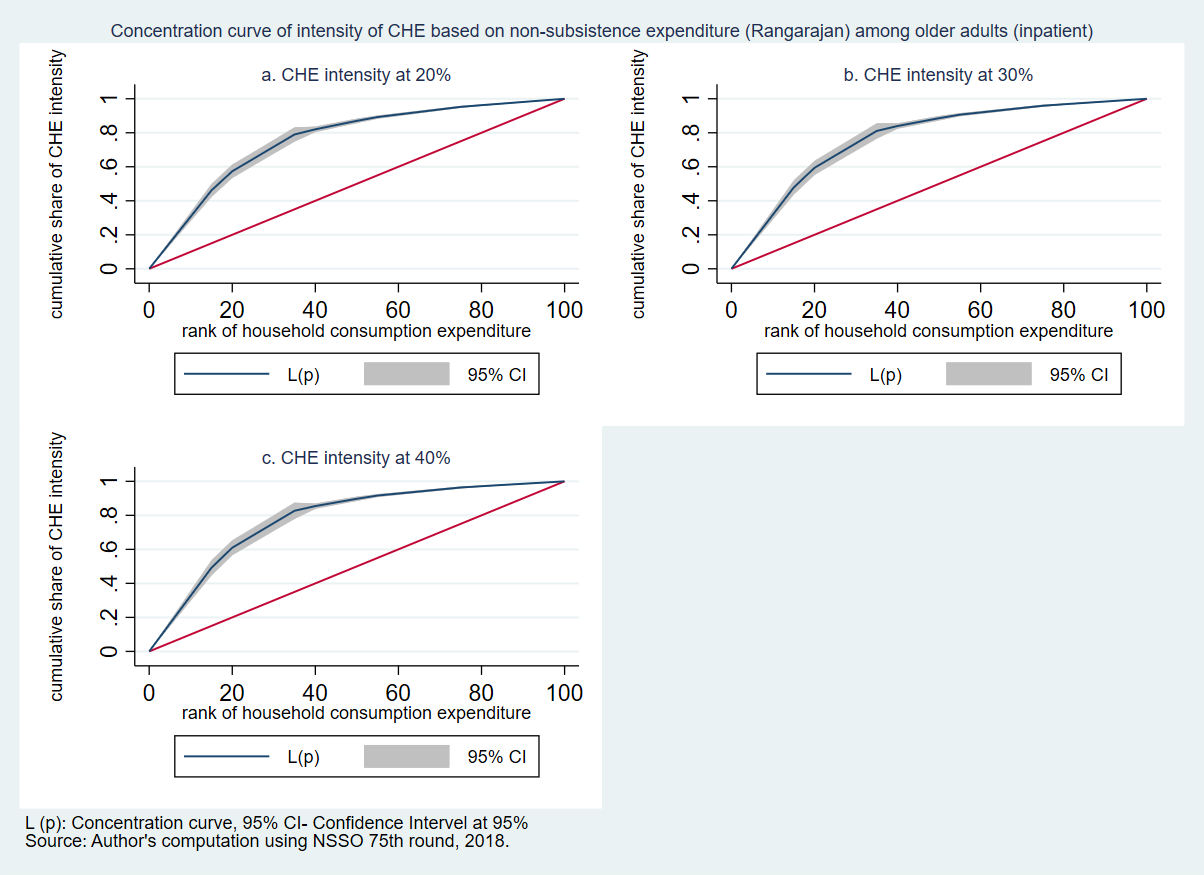
**

**Figure 15: Concentration curve of incidence of CHE based on subsistence expenditure among older adults (outpatient).**


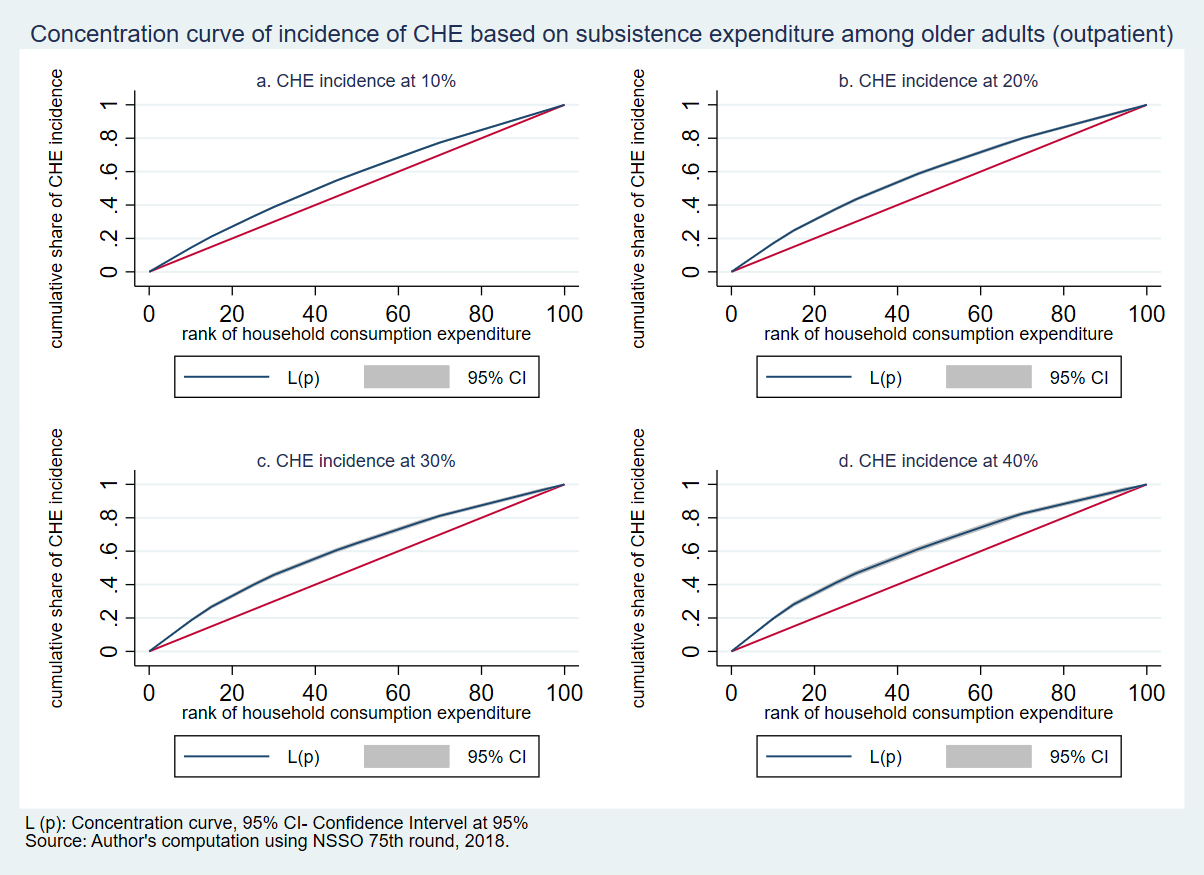


**Figure 16: Concentration curve of intensity of CHE based on subsistence expenditure among older adults (outpatient).**


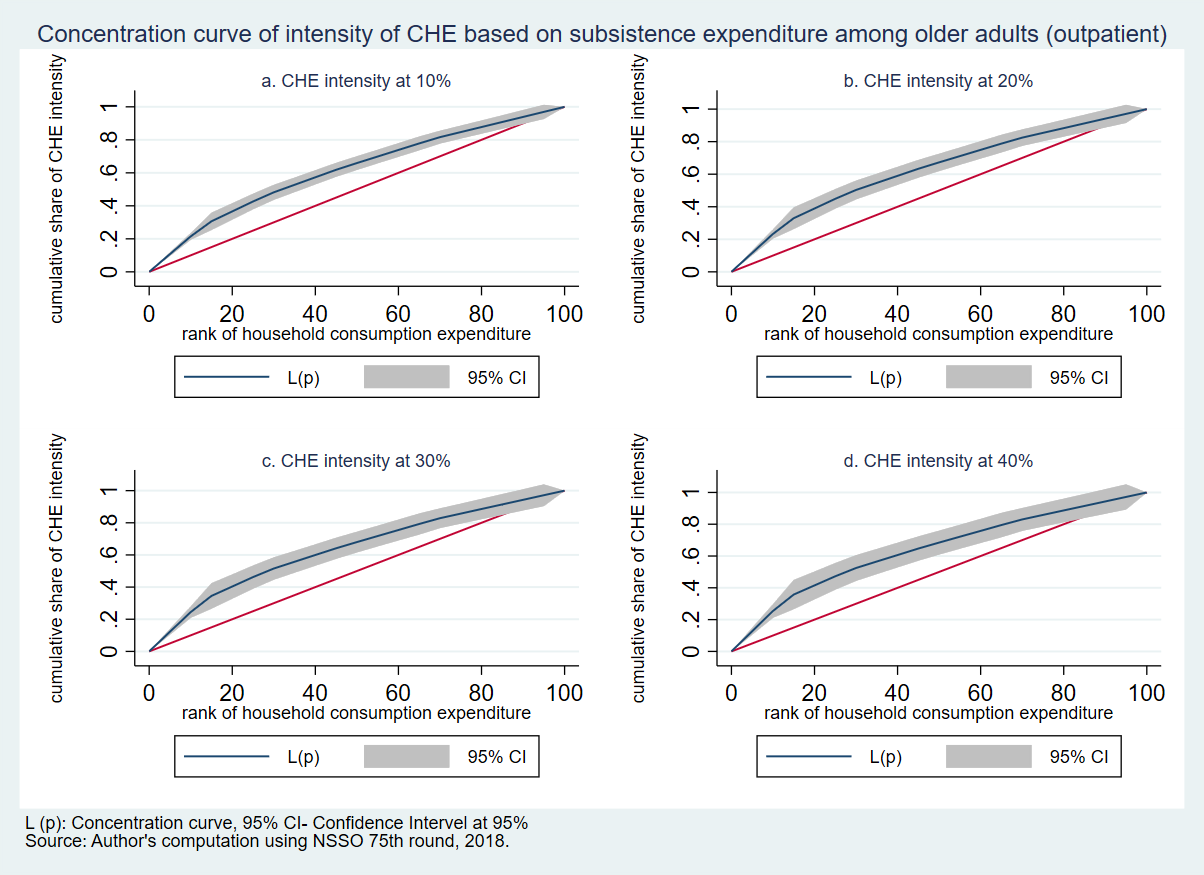


**Figure 17: Concentration curve of incidence of CHE based on non-subsistence expenditure (Tendulkar) among older adults (outpatient).**


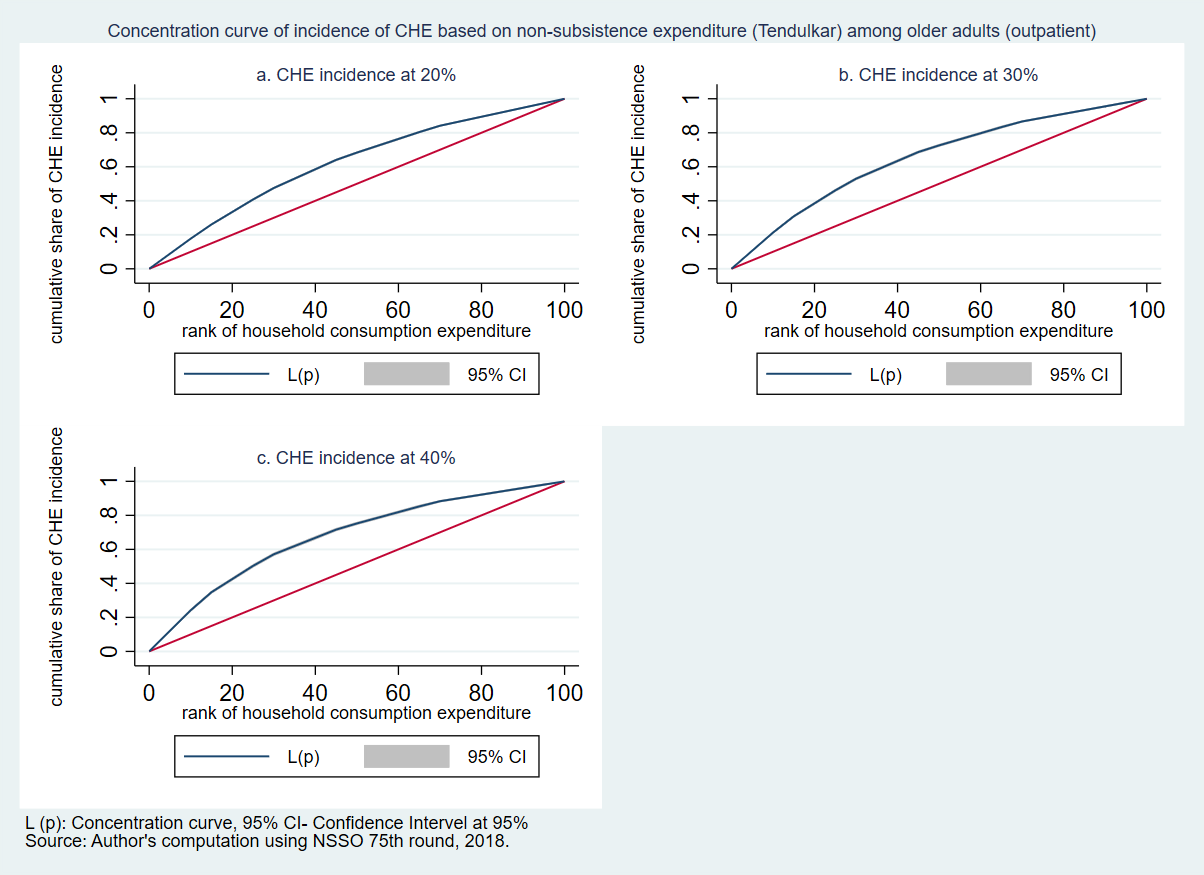


**Figure 18: Concentration curve of intensity of CHE based on non-subsistence expenditure (Tendulkar) among older adults (outpatient).**


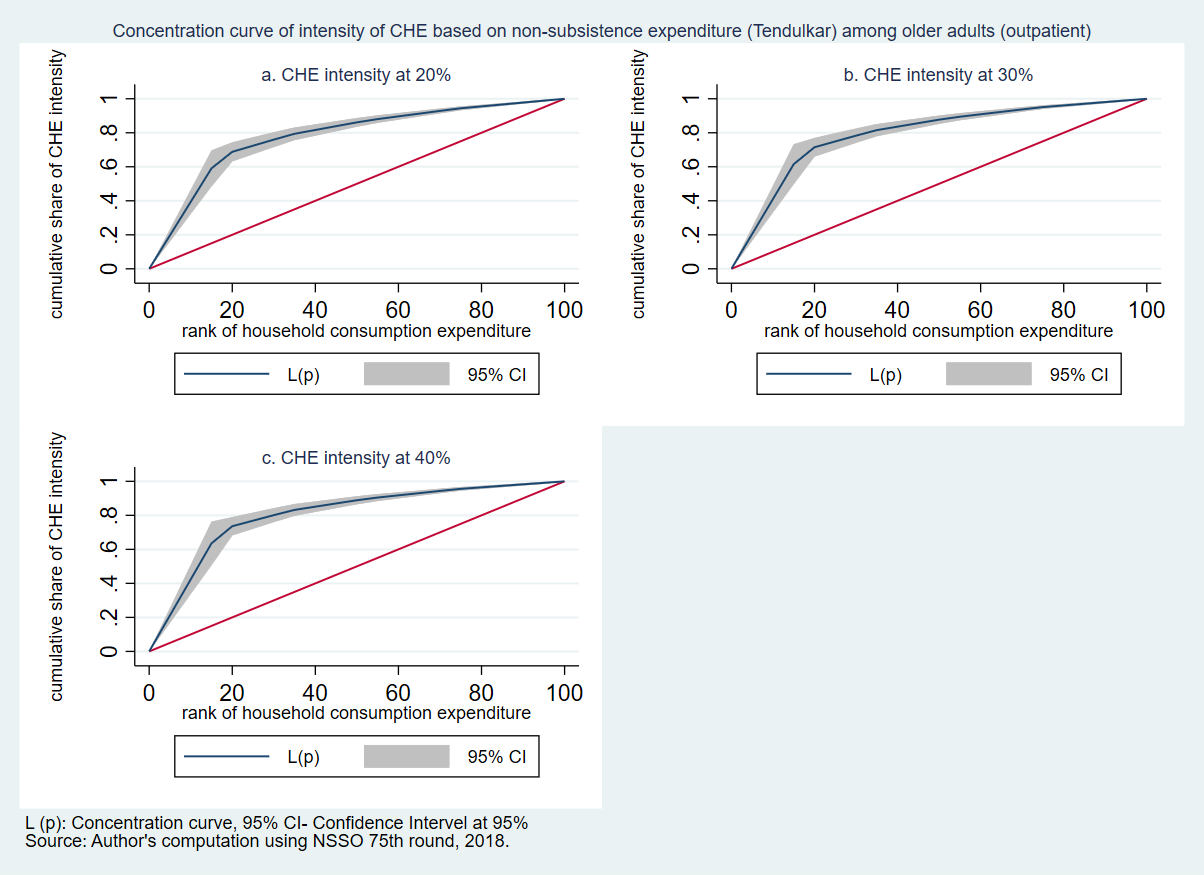


**Figure 19: Concentration curve of incidence of CHE based on non-subsistence expenditure (Rangarajan) among older adults (outpatient).**


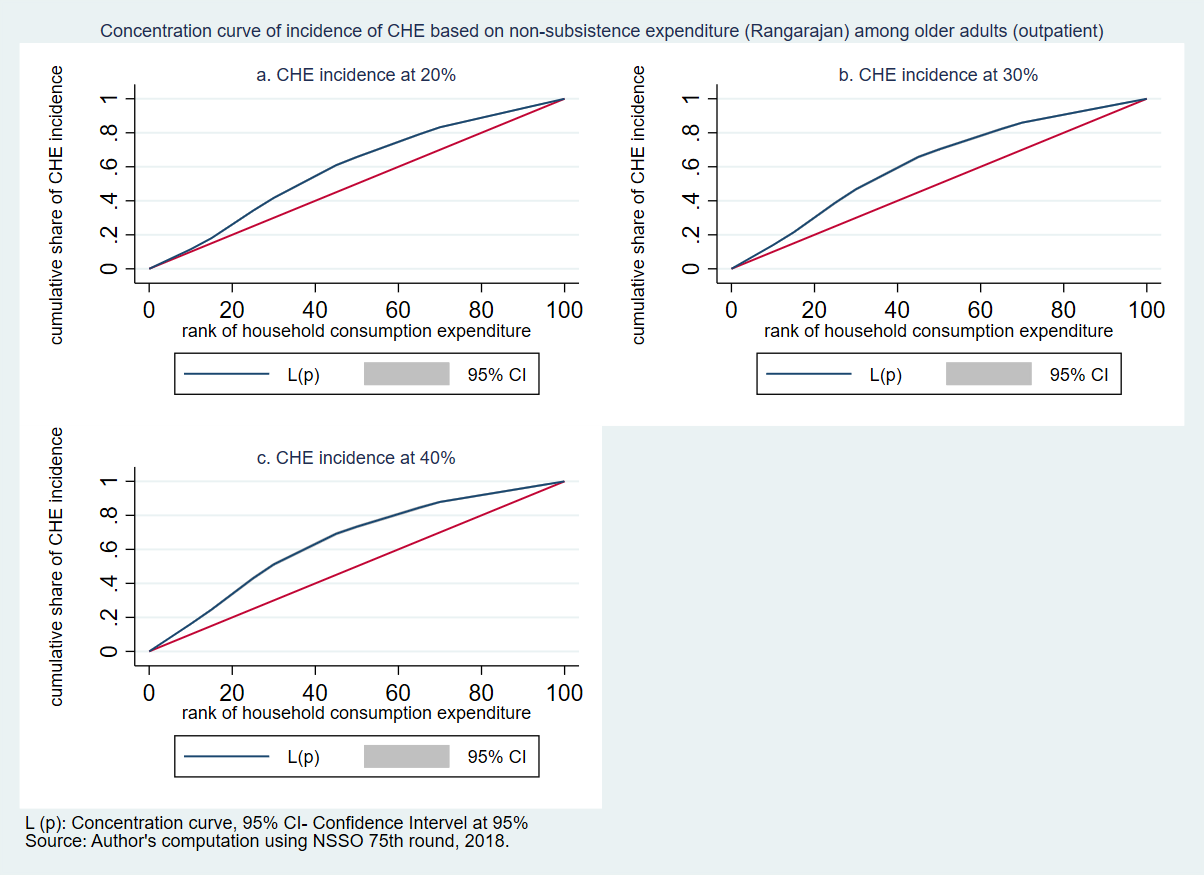


**Figure 20: Concentration curve of intensity of CHE based on non-subsistence expenditure (Rangarajan) among older adults (outpatient).**


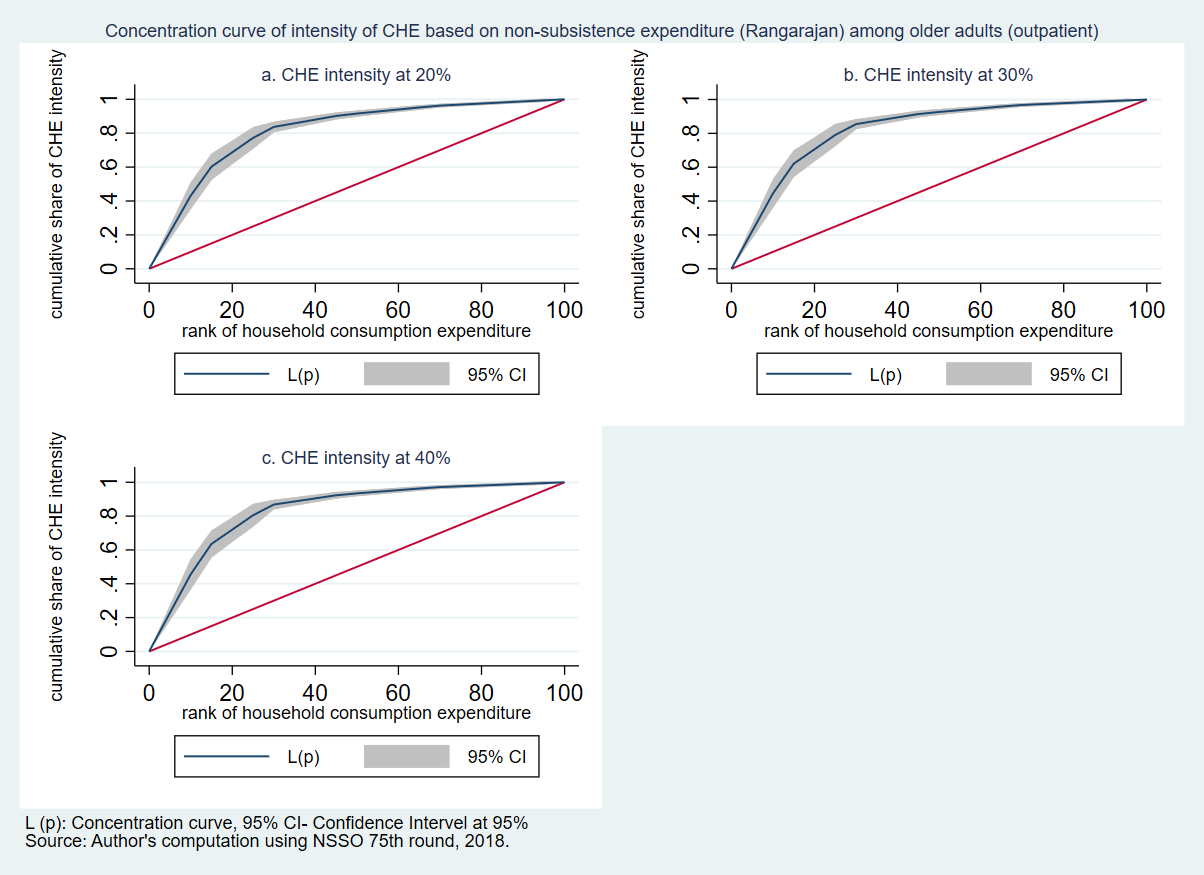

Supplement: Supplementary file 2 — Supplementary Information 2. [file 41598_2024_55142_MOESM2_ESM.docx]
